# Complex RNA Secondary Structures Mediate Mutually Exclusive Splicing of Coleoptera *Dscam1*

**DOI:** 10.3389/fgene.2021.644238

**Published:** 2021-03-30

**Authors:** Haiyang Dong, Lei Li, Xiaohua Zhu, Jilong Shi, Ying Fu, Shixin Zhang, Yang Shi, Bingbing Xu, Jian Zhang, Feng Shi, Yongfeng Jin

**Affiliations:** MOE Laboratory of Biosystems Homeostasis, Protection and Innovation Center for Cell Signaling Network, College of Life Sciences, Zhejiang University, Hangzhou, China

**Keywords:** clade-specific, Coleoptera, mechanism, RNA secondary structure, *Dscam1*, alternative splicing

## Abstract

Mutually exclusive splicing is an important mechanism for expanding protein diversity. An extreme example is the Down syndrome cell adhesion molecular (*Dscam1*) gene of insects, containing four clusters of variable exons (exons 4, 6, 9, and 17), which potentially generates tens of thousands of protein isoforms through mutually exclusive splicing, of which regulatory mechanisms are still elusive. Here, we systematically analyzed the variable exon 4, 6, and 9 clusters of *Dscam1* in Coleoptera species. Through comparative genomics and RNA secondary structure prediction, we found apparent evidence that the evolutionarily conserved RNA base pairing mediates mutually exclusive splicing in the *Dscam1* exon 4 cluster. In contrast to the fly exon 6, most exon 6 selector sequences in Coleoptera species are partially located in the variable exon region. Besides, bidirectional RNA–RNA interactions are predicted to regulate the mutually exclusive splicing of variable exon 9 of *Dscam1*. Although the docking sites in exon 4 and 9 clusters are clade specific, the docking sites-selector base pairing is conserved in secondary structure level. In short, our result provided a mechanistic framework for the application of long-range RNA base pairings in regulating the mutually exclusive splicing of Coleoptera *Dscam1*.

## Introduction

Alternative splicing is an important precursor RNA processing method to increase protein diversity in eukaryotes (Nilsen and Graveley, 2010; Pandey et al., 2020; Suresh et al., 2020). Alternative splicing is ubiquitous in various processes such as human nerve development, spermatogenesis, muscle contraction, and immune defense (Gallego-Paez et al., 2017). Abnormal alternative splicing events might be associated with diseases, e.g., cancers and neurodegenerative diseases (Kim et al., 2018; Montes et al., 2019; Bonnal et al., 2020; Wang et al., 2020). Pre-messenger RNA (pre-mRNA) alternative splicing has recently been thought to be related to the aging process and longevity (Bhadra et al., 2020). There are five main types of alternative splicing, including intron retention, exon skipping, alternative 3′ splice sites, alternative 5′ splice sites, and mutually exclusive splicing (Nilsen and Graveley, 2010; Zhang et al., 2016; Hatje et al., 2017; Jin et al., 2018). Mutually exclusive splicing is a specific type of alternative splicing; in a tandem exon array, only one variable exon can be spliced into the mature mRNA at a time (Smith, 2005). Mutually exclusive exons originate from exon duplication events (Graveley et al., 2004; Chen et al., 2011; Brites et al., 2013; Hatje and Kollmar, 2013; Yue et al., 2017). An extreme case of mutually exclusive splicing event is *Dscam1* in arthropods (Lee et al., 2010). In *Drosophila melanogaster*, *Dscam1* contains four clusters of variable exons 4, 6, 9, and 17 with 12, 48, 33, and 2 variable exons, respectively, and potentially produce 38,016 protein isoforms *via* mutually exclusive splicing (Schmucker et al., 2000). Due to the fact that homologous Dscam1 protein isoforms mediate self-avoidance (Wojtowicz et al., 2004; Soba et al., 2007; Zipursky and Grueber, 2013), such a staggering number of Dscam1 protein isoforms are functional for *D. melanogaster* neurons to identify self or non-self (Hattori et al., 2007; Hughes et al., 2007; Matthews et al., 2007; Kise and Schmucker, 2013). Dscam1 also plays an important role in the neuron circuit as an axon guidance receptor (Schmucker et al., 2000; Cvetkovska et al., 2013). Besides, evidence has revealed that Dscam1 is required for the immune function as the Ig superfamily member (Dong et al., 2006; Armitage et al., 2015; Ng and Kurtz, 2020).

An attractive regulatory mechanism of alternative splicing is the competitive RNA secondary structure mediating the splicing of exon variants (Graveley, 2005; Anastassiou et al., 2006; Yang et al., 2011; Xu et al., 2020). The most typical gene of this model is the variable exon 6 cluster of *Dscam1* in *D. melanogaster*. In the exon 6 cluster, two types of conserved intron elements participate in the alternative splicing of variable exon 6. The first intron element was located in the intron between the constitutive exon 5 and variable exon 6.1 and was referred to as the docking site. The docking site was the most conserved intron element in the entire *Dscam1* gene. Another type of intron element is the selector sequence; 48 selector sequences were located upstream of 48 variable exon 6s and were relatively conserved. Moreover, all 48 selector sequences were complementary to the only one docking site (Graveley, 2005). Besides, there is a class of heterogeneous nuclear ribonucleoprotein protein (hrp36) that uniformly covers the entire variable exon 6 cluster to maintain the fidelity of the mutually exclusive splicing (Olson et al., 2007). When the docking site pairs with the selector sequence of a specific exon to form an RNA secondary structure, the hrp36 protein on this exon will fall off, thereby promoting the splicing of this exon 6 (Graveley, 2005; Xu et al., 2019). Only the variable exon that forms secondary structures can release the inhibition proteins and trigger splicing. Moreover, an RNA locus control region (LCR) exists between constitutive exon 5 and the exon 6 docking site of *Dscam1* to promote the splicing of the adjacent downstream variable exon that forms the RNA secondary structure (Wang et al., 2012). Besides, similar docking site-selector base pairings also exist in vertebrate genes (Pervouchine et al., 2012; Suyama, 2013).

The mechanism by which competitive RNA secondary structure regulates the mutually exclusive splicing of variable exon 6 had been widely recognized (May et al., 2011). However, there are still some obstacles and doubts for the complete cognition of the variable exon 4 and 9 clusters of *Dscam1*. In our previous studies, downstream RNA pairings have been identified to regulate the splicing of exons 4 and 9 variants of *Dscam1* in *Drosophila* (Yang et al., 2011). Bidirectional competitive RNA secondary structure regulated the inclusion of variable exons in the exon 4 cluster of Hymenopteran *Dscam1* and the exon 9 clusters of Lepidopteran and Hymenopteran *Dscam1* (Yue et al., 2016). However, some other researchers questioned the regulatory mechanisms by which long-range competitive RNA secondary structure regulates the splicing of exons 4 and 9 due to the lack of apparent conserved intron elements (Haussmann et al., 2019; Ustaoglu et al., 2019). Recently, a unique evolutionary midge-specific docking site has been found in the exon 6 cluster, which regulates the process of alternative splicing *via* base pairing (Hong et al., 2020). However, the splicing of exon 4 and 9 clusters has still not been well explained.

Whether clade- or species-specific but RNA secondary structure conserved docking site can mediate alternative splicing of exons 4 and 9 of *Dscam1*? We focus on Coleoptera to further explore that. Coleoptera, roughly 360,000 described species make up about 40% of all insect species (Bouchard et al., 2017), is the largest order in Insecta (Woodcock et al., 2013; Zhang et al., 2018), and make up almost 25% of all animals (Hunt et al., 2007). Thus, many species provide convenience for evolutionary analysis. Moreover, the rapid development of public databases has enabled the genomic data of multiple species of Coleoptera to be found in GenBank (Bocak et al., 2014), providing us with a rich source of sequence alignment. These characteristics make Coleoptera a suitable material for studying alternative splicing of *Dscam1*.

Through sequence alignment and secondary structure prediction, we found that the clade-specific docking site can mediate the selection of exon 4 *via* the formation of RNA secondary structure with the selector sequences in a base-pairing manner. Moreover, bidirectional competitive RNA secondary structures were also discovered in the exon 9 cluster. Although the primary sequence of exon 4 and 9 docking sites were clade specific or species specific, the docking site-selector base pairing was conserved in the RNA secondary structure level. In addition, due to the short intron of the exon 6 cluster in Coleoptera, most selector sequences were partially located in exon regions. Taken together, our findings provided a mechanistic framework that competitive RNA secondary structure regulates mutually exclusive splicing of *Dscam1* exon 4, 6, and 9 clusters in Coleoptera.

## Materials and Methods

### Identification and Annotation of *Dscam1* Gene Structure

The *Dscam1* genome sequences of Coleoptera species were obtained by using the *Dscam1* of *D*. *melanogaster* as the query sequences and performing TBLASTN search in the NCBI WGS database^1^. Annotation of the *Dscam1* was performed by comparative genomics with cross-species or intraspecies. The identification and the numbers of variable exons 4, 6, 9, and 17 were confirmed by nucleic acid or protein sequence alignment of variable exons between different species or within species. Combined with the existing RNA sequencing data, the boundaries of the variable exons can be further confirmed (Supplementary Table 1).

### Sequence Alignment and Secondary Structure Analysis

Clustal Omega^2^ was applied to sequence alignment. The docking site-selector sequences base pairings were predicted by the Mfold project^3^ (Zuker, 2003). The conserved selector sequences were derived *via* the WebLogo^4^ (Crooks et al., 2004).

### The Drawing of the Evolutionary Tree

The amino acid sequence was composed of constitutive exons and randomly selected variable exons in each cluster, and the amino acid sequences of 14 Coleopteran *Dscam1* were imported into MEGA X^5^. Evolutionary relationships of taxa were drawn based on the Minimum Evolution method (Kumar et al., 2018).

## Results

### *Dscam1* Gene Structure and Molecular Diversity in Coleoptera Species

**FIGURE 1:**
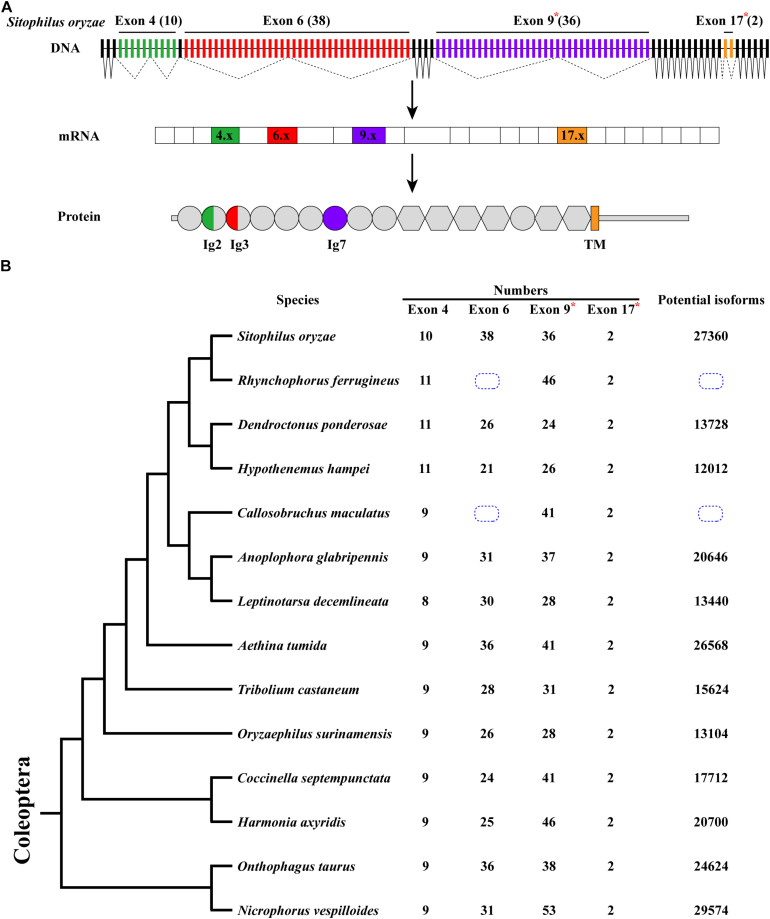
*Dscam1* gene structure and molecular diversity of Coleoptera species. **(A)** Schematic diagram of the *Dscam1* gene structures of *S*. *oryzae*. Variable exons are marked by colored boxes, constitutive exons as black boxes. Dscam1 protein includes 10 immunoglobulin (Ig) domains (circles), six fibronectin type III domains (hexagons), one TM domain, and cytoplasmic tails. The variable exons 4 and 6 encode half Ig2 and Ig3 domains, respectively, while exons 9 and 17 encode the whole Ig7 and transmembrane domains, respectively. Variable exon 11 and 24 clusters of *S*. *oryzae* are evolutionarily homologous to exon 9 and 17 clusters of *D*. *melanogaster* and are marked with an “*” and named exons 9 and 17. **(B)** A phylogenetic tree of Coleoptera species is shown on the left. Evolutionary relationships of taxa were drawn with MEGA X. The number of variable exons in each cluster is shown in the middle, and the total potential isoforms are shown on the right. The blue dotted line box indicates that the number of exon 6 cannot be defined.

*Sitophilus oryzae*, a representative species of Coleoptera, has a similar gene structure to *D*. *melanogaster Dscam1*, containing 26 constitutive exons and 4 clusters of variable exons. However, the number of variable exons in exon 4, 6, and 9 clusters were different from those in *D*. *melanogaster* (Schmucker et al., 2000). In *S*. *oryzae*, exon 4, 6, 9, and 17 clusters contain 10, 38, 36, and 2 variable exons, respectively. It potentially produces 27,360 (10 × 38 × 36 × 2) protein isoforms through mutually exclusive splicing. Dscam1 protein contains 10 immunoglobulin (Ig) domains and six fibronectin type III (FNIII) domains, a transmembrane domain, and a C-terminal intracellular region. Variable exons 4 and 6 encode half Ig2 and Ig3 domains, respectively, while exons 9 and 17 encode the whole Ig7 and transmembrane domains, respectively (Figure 1A).

After annotation of *Dscam1* genes in other 12 species (*Rhynchophorus ferrugineus*, *Dendroctonus ponderosae*, *Hypothenemus hampei*, *Callosobruchus maculatus*, *Anoplophora glabripennis*, *Leptinotarsa decemlineata*, *Aethina tumida*, *Oryzaephilus surinamensis*, *Coccinella septempunctata*, *Harmonia axyridis*, *Onthophagus taurus*, and *Nicrophorus vespilloides*), we found that the transmembrane domain of each species contains two variable exons (exon 17). However, the number of variable exons in exon 4, 6, and 9 clusters of the Coleoptera species vary. The number of variable exon 4s ranges from eight to 11, mostly with 9 exon variants, and does not change as much as exons 6 and 9. In the exon 4 cluster, the variable exon 4.4 was missing during evolution, resulting in only eight variants in *L*. *decemlineata*. On the contrary, 10 or 11 variable exons can be identified due to the duplication of variable exons in the *S. oryzae*, *R. ferrugineus*, *D. ponderosae*, and *H. hampei*, which all belong to the same superfamily (Supplementary Figures 1, 2). Correspondingly, the number of variable exon 9 ranges to a staggering 53 in *N*. *vespilloides*, more than twice to that in *D*. *ponderosae*, which only have 24. In the exon 6 cluster, unfortunately, due to the genomic sequence break in the database, we failed to determine the number of exon 6 variants of *Dscam1* in *C*. *maculatus* and *R*. *ferrugineus*. However, an interesting phenomenon was that the number of variable exons of *S*. *oryzae* was nearly twice that of *H*. *hampei*, even if they belong to the same family. Moreover, the number of exon 6s of all analyzed species was much smaller than the *D*. *melanogaster*, which has 48 exon 6 variants (Figure 1B). Although the number of variable exons varies between different species, *Dscam1* of most Coleoptera species can potentially generate tens of thousands of protein isoforms (the potential protein isoforms of *R. ferrugineus* and *C. maculatus Dscam1* are uncertain due to the lack of genomic sequence in exon 6 clusters).

### Downstream RNA Pairing Mediates Mutually Exclusive Splicing of Exon 4 Cluster

**FIGURE 2:**
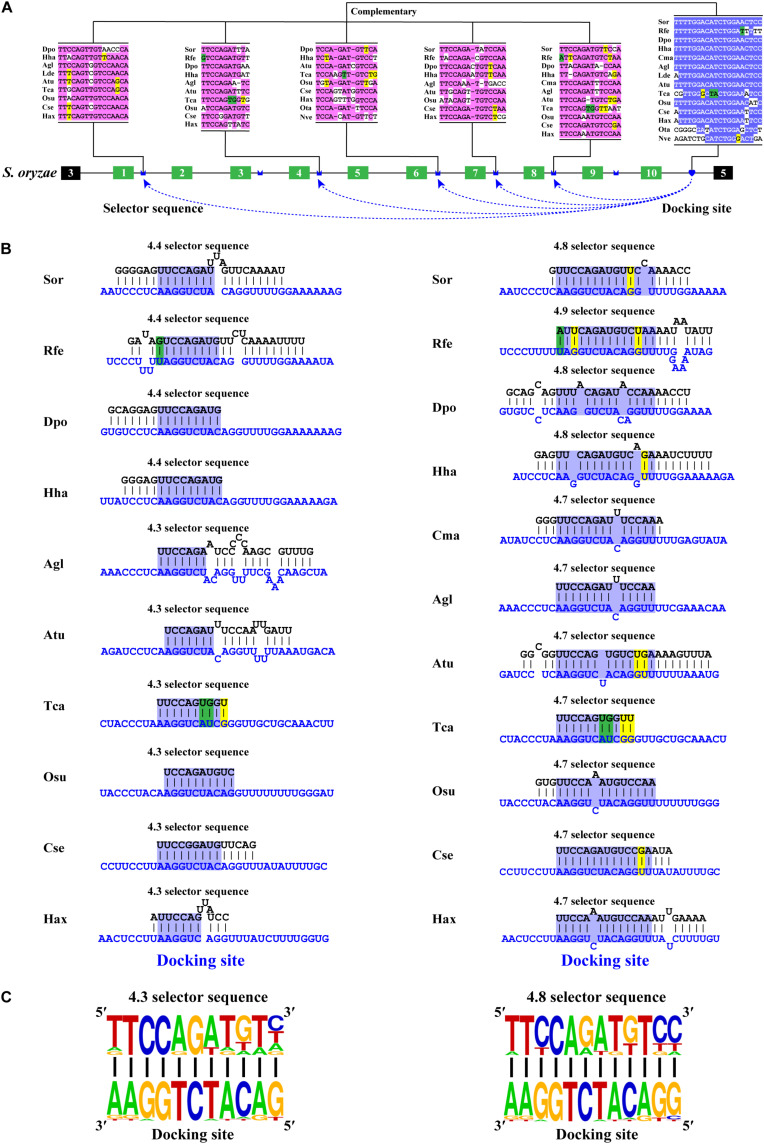
Conserved downstream RNA pairings mediate mutually exclusive splicing of *Dsacm1* exon 4. **(A)** Schematic diagram of the *Dscam1* exon 4 of *S*. *oryzae*. The docking site (marked by blue heart) and each selector sequence (marked by blue crowns) are complementary. The conserved nucleotide sequences of the docking site and selector are highlighted in different colors. The base sequences are shown from 5′ to 3′. Abbreviations of the species name are shown in Supplementary Table 1. **(B)** The RNA secondary structures between the docking site and 4.3 and 4.8 selector sequences are shown among Coleoptera species. The sequences that make up the core region of the RNA secondary structure are highlighted in blue. The selector sequences are shown in black font, and the docking sites are shown in blue font. Nucleotides of compensatory structural covariations that maintain the base pairing are shaded in green, and their evolutionary intermediates (U-G, G-U) are shaded in yellow. **(C)** The most frequent nucleotides at each position of the 4.3 and 4.8 selector sequences among species are complementary to the docking site.

Phylogenetic analyses revealed that most variable exon 4s were orthologous in Coleoptera species (Supplementary Figures 1, 2), indicating that the variable exon 4s derived from the common ancestor and less exon duplication or loss occur during the evolutionary process. This was consistent with the previous studies, which suggested that most exon 4s might be orthologous in the insects (Lee et al., 2010). To decipher the mechanism for *Dscam1* exon 4 mutually exclusive splicing, we first searched the conserved intron element. Docking site-selector sequence base pairing mediating mutually exclusive splicing in *Dscam1* exon 4 has been identified in *Drosophila* and Hymenoptera species (Yang et al., 2011; Yue et al., 2016). However, the primary sequences of the docking sites between *Drosophila* and Hymenoptera species were different. Therefore, we speculated that the primary sequences of the docking site in the exon 4 cluster were evolutionarily specific in the Coleoptera species. Through sequence alignment, we found a conserved intron element (docking site) downstream of the last variable exon 4 (Figure 2A). Indeed, the docking site sequences in Coleoptera were different from *Drosophila* and Hymenoptera species, indicating a clade-specific docking site in Coleoptera species. Moreover, only one apparent docking site has been found, similar to the exon 4 cluster of *Drosophila*, while there was a docking site on both sides of the exon 4 cluster in Hymenoptera species (Yue et al., 2016). Through RNA secondary structure prediction, evolutionarily conserved selector sequences complementary to the docking site were identified, and all the selector sequences were located downstream of the variable exons (Figures 2B,C and Supplementary Figures 3–5). Moreover, clear evidence of compensatory structural covariations and evolutionary intermediates exist within the core region of the RNA secondary structure formed by docking site-selector base pairing (Figures 2A,B). Due to the distant evolutionary relationship, the docking sites in *O*. *taurus* and *N*. *vespilloides* were less conserved compared to that in other species. However, conserved RNA secondary structures within these species were found (Supplementary Figure 6). Taken together, these results suggested that the downstream RNA base pairing could mediate the mutually exclusive splicing of variable exon 4 cluster, and the docking site showed to be clade specific.

### Most Selector Sequences of Exon 6 Cluster Are Partially Located in Variable Exon Region

**FIGURE 3:**
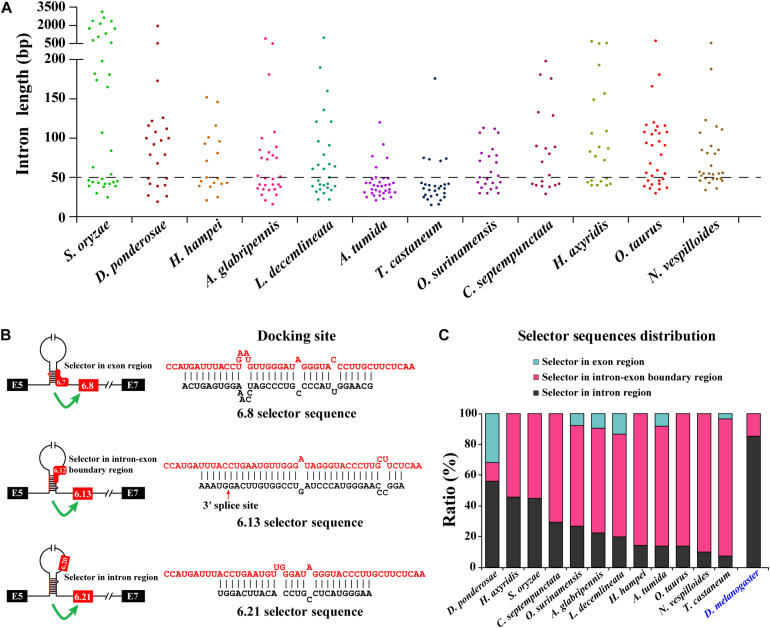
Most exon 6 selector sequences are partially located in the exon sequence of Coleoptera *Dscam1* exon 6. **(A)** The intron length between two variable exon 6s of *Dscam1* in Coleoptera species is shown. **(B)** Three types of the location of selector sequences and the corresponding secondary structures are shown in *A*. *tumida*. The selector sequences are shown in black font, and the docking sites are shown in red font. **(C)** Comparison of the distribution of exon 6 selector sequences between Coleoptera species and *D*. *melanogaster*.

After annotating the exon 6 cluster of *Dscam1* in Coleoptera, we calculated the length of introns between two variable exons. Surprisingly, up to 82% of intron lengths were <150 bp. More interestingly, more than 45% of intron lengths were <50 bp (Figure 3A). Due to the small intron (<50 bp), maybe nearly half of the selector sequences will be located in the exon region to avoid the steric hindrance. To test our hypothesis, we identified the evolutionarily conserved docking site of exon 6 cluster through sequence alignment and marked the selector sequences located upstream of each variable exon 6 by long-range competitive RNA secondary prediction. Notably, almost all exon 6s could find the corresponding selector sequence (Supplementary Figures 7, 8). These results indeed illustrated and consolidated that the mechanism of mutually exclusive splicing of the exon 6 cluster was regulated by the competitive RNA secondary structure. Moreover, it suggested that we may have found the correct selector sequences.

To explore the distribution of the selector sequences, we divided the location of the selector sequences into three types: completely located in the exon region, located in the intron–exon boundary region, and completely located in the intron region (Figure 3B). In the exon 6 cluster of *D*. *melanogaster Dscam1*, 85% (41 out of 48) of the selector sequences were completely located in intron regions, while the remaining seven selector sequences were located in the intron–exon boundary region (Figure 3C; Graveley, 2005). On the contrary, after analyzing the distribution of exon 6 selector sequences of 12 Coleoptera species, we found that 56% (14 out of 25) of the selector sequences of *D. ponderosae* exon 6 were completely located in intron regions, 12% (three out of 25) of the selector sequences were completely located in the exon region, and 32% (eight out of 25) of the selector sequences were located in the intron–exon boundary region. More obviously, only 7% (two out of 28) of the selector sequences of *T. castaneum* exon 6 completely located in intron regions, while 26 out of 28 selectors included the exon sequences (Figure 3C). In conclusion, our discovery in the exon 6 cluster of Coleoptera *Dscam1* expanded our understanding that the selector sequences can be located in or included the variable exon sequence, not just in the intron region.

### Dual RNA Pairing Mediates Mutually Exclusive Splicing of Exon 9 Cluster

**FIGURE 4:**
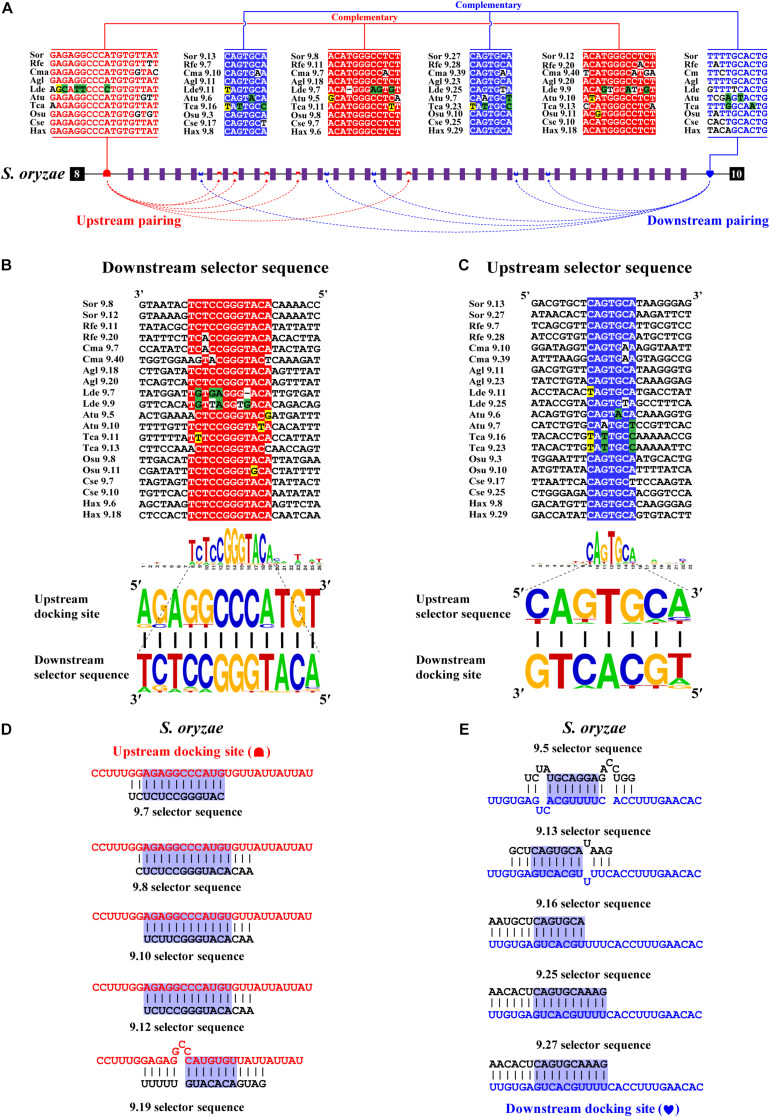
Conserved dual docking site and selector sequences base pairing of Coleoptera *Dscam1* exon 9. **(A)** Schematic diagram of the *Dscam1* exon 9 of *S*. *oryzae*. Constitutive exons are depicted as black boxes and variable exon 9 as purple boxes. Upstream docking site (marked by red semicircles) and downstream docking site (marked by blue heart) complementary to the downstream selector sequences (marked by red saddle shapes) and upstream selector sequences (marked by blue crowns), respectively. The dashed arrow represents the RNA–RNA interaction of upstream or downstream base pairings. The most frequent nucleotides at upstream and downstream docking sites are depicted in red and blue, respectively, while the most frequent nucleotides at the selectors are depicted in red and blue, respectively. The base sequences are shown from 5′ to 3′. **(B,C)** Upstream and downstream selector sequences alignment. The core regions of the downstream or upstream selector sequences are highlighted red or blue, respectively. The most frequent nucleotides at each position of the downstream or upstream selector sequences are complementary to the upstream or downstream docking sites, respectively. Nucleotides of compensatory structural covariations that maintain the base pairing are shaded in green, and their evolutionary intermediates (U-G, G-U) are shaded in yellow. **(D,E)** The secondary structures between upstream or downstream base pairing are shown in *S*. *oryaze*. The sequences that make up the core region of the stem are highlighted in blue. The upstream and downstream selector sequences are shown in black font; upstream and downstream docking sites are shown in red and blue fonts, respectively.

Next, we decoded the mutually exclusive splicing mechanism of the *Dscam1* exon 9 cluster. Previous studies have reported that the unidirectional-competitive RNA secondary structure regulates splicing of *Dscam1* exon 9 in *Drosophila* (Yang et al., 2011), bidirectional RNA base pairing in Lepidoptera, and Hymenoptera *Dscam1* exon 9 (Yue et al., 2016). What is more, the primary sequences of the docking site showed to be clade specific between *Drosophila*, Lepidoptera, and Hymenoptera. Likewise, through genome sequence alignment and RNA secondary structure prediction, two intron elements (upstream docking site and downstream docking site) in the exon 9 cluster were found. However, 10 out of 14 chosen species shared a conserved upstream docking site (Figure 4A); the primary sequence of upstream docking sites in *D*. *ponderosae*, *H*. *hampei*, *O*. *taurus*, and *N*. *vespilloides* was specific (shown later). By contrast, the downstream docking sites were conserved in 14 species. Moreover, both the primary sequences of upstream or downstream docking sites were clade specific compared to that of Lepidoptera and Hymenoptera.

Through further RNA secondary structure prediction, many downstream selector sequences complementary to the upstream docking site and many upstream selector sequences complementary to the downstream docking site were identified (Supplementary Figures 9–14). However, due to the poor homology between the variable exon 9s of *Dscam1* in Coleoptera species, it was difficult to confirm the conservativeness of evolutionarily corresponding selector sequences. Alternatively, we selected two selector sequences paired with upstream or downstream docking sites in each species. Through the alignment of so many downstream and upstream selector sequences, respectively, the core area of downstream and upstream selector sequences can form base pairing to upstream and downstream docking sites, respectively (Figures 4B,C). Moreover, compensatory structural covariations and evolutionary intermediates were shown to be formed by docking site-selector base pairing (Figures 4A,B). Upstream and downstream base pairings can form a relatively strong remote competitive RNA secondary structure (Figures 4D,E). Therefore, we concluded that clade-specific upstream and downstream docking sites regulated the mutually exclusive splicing of the *Dscam1* exon 9 cluster in Coleoptera species.

### The Primary Structure of the Docking Site Is Specific, but the RNA Secondary Structure Is Conserved

**FIGURE 5:**
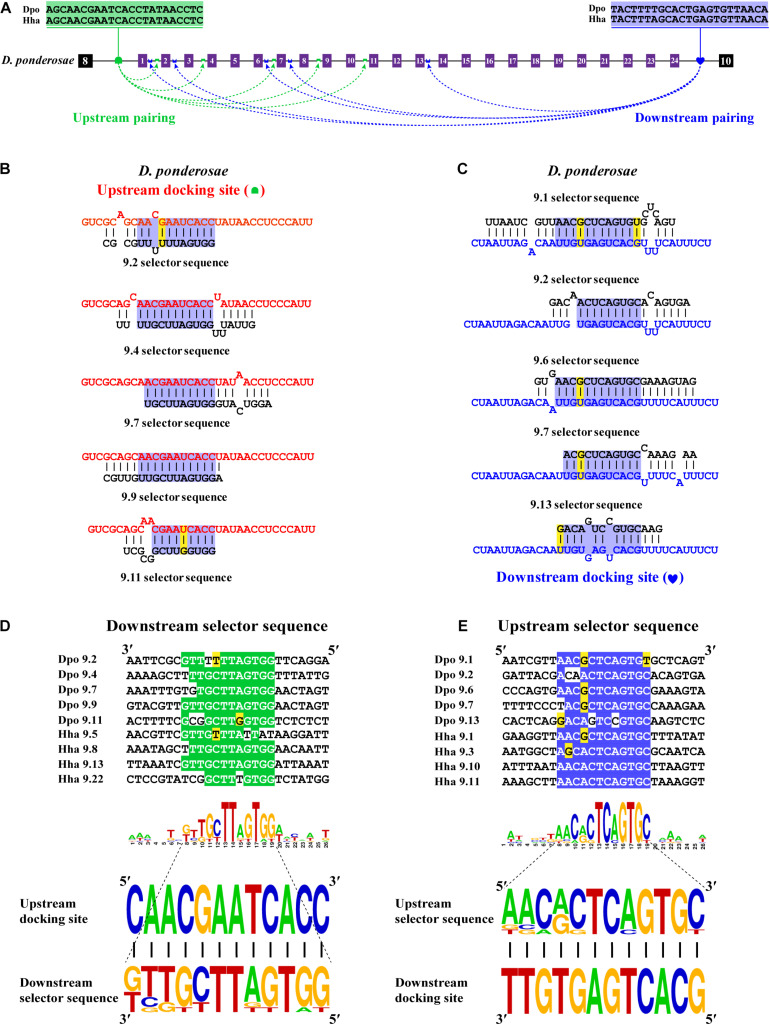
Species-specific upstream docking site in *D*. *ponderosae* and *H*. *hampei Dscam1* exon 9. **(A)** Schematic diagram of the *Dscam1* exon 9 of *D*. *ponderosae*. Upstream docking site (marked by green semicircles) and downstream docking site (marked by blue heart) complementary to the downstream selector sequences (marked by green saddle shapes) and upstream selector sequences (marked by blue crowns), respectively. The dashed arrow represents the RNA–RNA interaction of upstream or downstream pairing. The most frequent nucleotides at upstream and downstream docking sites are depicted in green and blue, respectively. The base sequences are shown from 5′ to 3′. **(B,C)** The secondary structures between upstream or downstream base pairing are shown in *D*. *ponderosae*. The sequences that make up the core region of the stem are highlighted in blue. The upstream and downstream selector sequences are shown in black font; upstream and downstream docking sites are shown in red and blue fonts, respectively. **(D,E)** Upstream and downstream selector sequences alignment. The core regions of the downstream or upstream selector sequences are highlighted green or blue, respectively. The most frequent nucleotides at each position of the downstream or upstream selector sequences are complementary to the upstream or downstream docking sites, respectively. Nucleotides of compensatory structural covariations that maintain the base pairing are shaded in green, and their evolutionary intermediates (U-G, G-U) are shaded in yellow.

Bidirectional competitive RNA secondary structure has been identified in the exon 9 cluster. However, in the process of intron sequence alignment, the upstream docking site of *D*. *ponderosae* and *H*. *hampei* showed specificity compared to the other 10 species, but they were evolutionarily conserved (Figure 5A). Recently, a midge-specific docking site in the exon 6 cluster has been identified (Hong et al., 2020). Therefore, we suspected that species-specific upstream docking sites existed in the exon 9 cluster of *D*. *ponderosae* and *H*. *hampei*. Through RNA secondary structure prediction, many downstream and upstream selector sequences were complementary to the upstream and downstream docking sites, respectively (Figures 5B,C and Supplementary Figure 15). Similarly, due to the poor evolutionary correspondence between variable exon 9s in *D*. *ponderosae* and *H*. *hampei*, we selected four selector sequences of each species for further analysis. Through selector sequences alignment, the upstream or downstream selector sequences shared a core conserved region, and the core region could interact with the upstream or downstream docking site *via* base pairing (Figures 5D,E). In addition, compensatory structural covariations and evolutionary intermediates exist within the core region of the RNA secondary structure formed by docking site-selector base pairing (Figures 5B–E). Likewise, the upstream docking sites were species specific, but the base pairings were conserved at the secondary structure level within species in *O*. *taurus* and *N*. *vespilloides* (Supplementary Figures 16, 17). However, all species shared a common region of downstream docking sites to form the downstream RNA base pairings. Hence, a species-specific docking site but with conserved RNA secondary structure could mediate alternative splicing of *Dscam1* exon 9.

### Summary of Bidirectional Competitive RNA Secondary Structure in Exon 9

**FIGURE 6:**
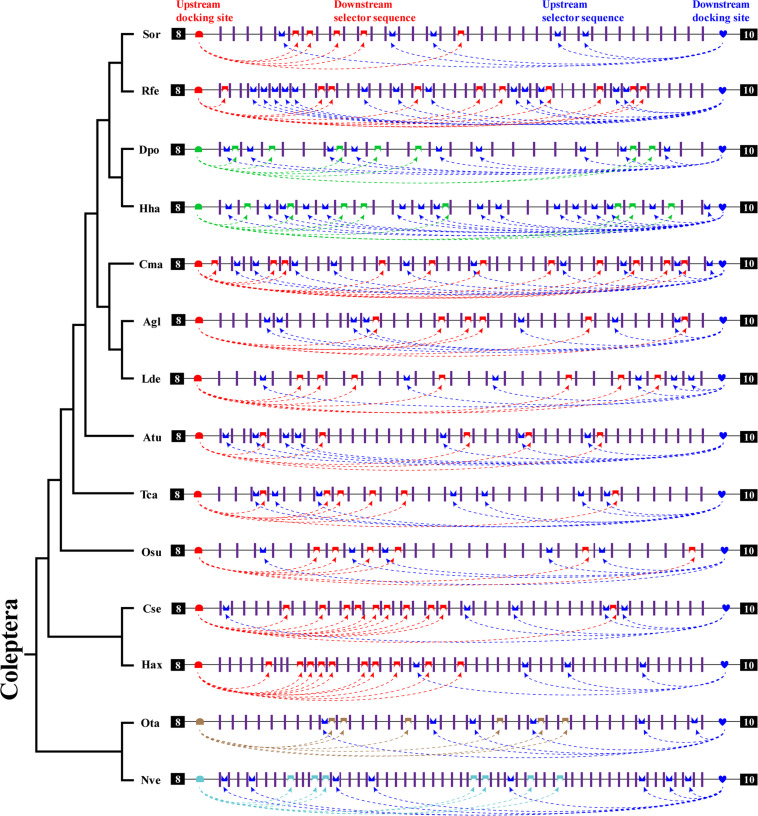
A summary of bidirectional RNA pairing of *Dscam1* exon 9 in Coleoptera species. Overview of the arrangement of the docking site and selector sequence of exon 9 cluster of Coleoptera *Dscam1*. Symbols used are the same as in Figure 4, and the exons, introns, docking sites, and selectors are not drawn to scale. Specific upstream or downstream docking sites are shown in different colors. The dashed arrow represents the RNA–RNA interaction of upstream or downstream pairing. The phylogenetic tree of Coleoptera species is shown on the left.

In this study, we identified bidirectional RNA base pairing in *Dscam1* exon 9 in Coleoptera species. Overall, 10 out of 14 chosen species shared a conserved upstream docking site, while the upstream docking site in *D*. *ponderosae*, *H*. *hampei*, *O*. *taurus*, and *N*. *vespilloides* was species specific. Besides, upstream docking sites between *D*. *ponderosae* and *H*. *hampei* were evolutionarily conserved. For the downstream base pairing, all chosen species shared a conserved downstream docking site (Figure 6). Taken together, we considered that during the evolution process, the primary sequences of the docking site would be mutated, but the base pairings in the secondary structure level were still conserved. Moreover, the dual docking sites may make up the splicing abnormality caused by the mutation of the docking site during evolution. Therefore, the bidirectional RNA secondary structure may be an adaptation of the organism to the evolution process.

## Discussion and Conclusion

Through the comparative analyses of 14 species in Coleoptera, We propose a potential mechanism that competing RNA secondary structure could mediate mutually exclusive splicing in Coleoptera *Dscam1*. Downstream base pairings directed the splicing of variable exon 4s. In the exon 6 cluster, we expanded the location of the selector sequence that may be located in the exon region. Moreover, species- or clade-specific docking sites could mediate the splicing of exon 9 by forming a bidirectional competitive RNA secondary structure. These studies have provided more evidence for the view that competitive RNA secondary structures mediate *Dscam1* alternative splicing from an evolutionary perspective.

The mutually exclusive alternative splicing model of *Dscam1* exon 6 cluster guided by competitive secondary structure was proposed as early as 2005. Even if it has undergone evolution for 300 million years, the docking site of the exon 6 cluster is conserved through the entire Insecta (Graveley, 2005). Recently, a midge-specific docking site but base-pairing conserved in secondary structure level in the exon 6 cluster has been found (Hong et al., 2020), indicating a species-specific docking site in the exon 6 cluster. Our study also predicted the secondary structure in the Coleoptera exon 6 cluster, and most selector sequences were partly located in the exons. This was different from the previous view and had a new inspiration for the identification of the selector sequence. Overall, the docking site of *Dscam1* exons 4 and 9 is clade or species specific and less conserved to exon 6. Therefore, less apparent docking sites make some researchers question the mechanism model of competitive RNA secondary structure regulating the alternative splicing of exons 4 and 9 clusters (Haussmann et al., 2019; Ustaoglu et al., 2019). In this study, through sequence alignment, we identified the clade- or species-specific docking sites of Coleoptera *Dscam1* exon 4 and exon 9 clusters, but the docking site-selector base pairings are conserved in the secondary structure level, which provided more evidence for *Dscam1* exon 4 and 9 clusters of competitive RNA secondary structure to regulate mutually exclusive alternative splicing.

We have used the Mfold program, which uses a minimum free energy algorithm, to perform RNA secondary structure prediction (Zuker, 2003). The prediction results were similar to some other programs, for example, RNAstructure, a program that calculates the base-pairing probabilities for RNA or DNA sequences by predicting the lowest free energy structures (Mathews et al., 2004), and RNAfold, a program that also uses the minimum free energy algorithm and has a partition function for computing base-pairing probabilities (Bompfunewerer et al., 2008). Although the competitive RNA secondary structures of Coleoptera *Dscam1* were shown in this paper, experimental verification of these predicted secondary structures is difficult due to the limitation of technical means. The main reasons are as follows: First, it is difficult to construct an expression vector due to the large size of the variable exon cluster (30,000 bp in *S. oryzae Dscam1* exon 9). Second, even if the minigene of the variable exon cluster was constructed, the variable exons may not be spliced normally (Graveley, 2005). Third, using the CRISPR-Cas9 system to directly perform mutation *in vivo* seems hard to carry out in practice due to the lack of model organisms in Coleoptera. However, it will be interesting if there are useful systems to solve the experimental verification problems in the future.

Coleopteran insects have not been thoroughly studied, and there is no established genetic manipulation system as mature as the model organism *D*. *melanogaster*. The experimental operation is difficult. Therefore, all the secondary structures and their effects described in this article are predicted. In the future, it is necessary to conduct systematic research on Coleoptera, explore its genetic research tools, and further experimentally verify the regulatory effect of our proposed RNA secondary structure on alternative splicing.

## Data Availability Statement

The original contributions presented in the study are included in the article/Supplementary Material, further inquiries can be directed to the corresponding author/s.

## Author Contributions

YJ conceived the project. HD, LL, JS, and YF found the sequence. LL, YF, SZ, and JS predicted the secondary structure. YS, YF, LL, and JS made the evolutionary tree. HD, LL, and JS drew the pictures. XZ, BX, JZ, and FS analyzed the data. HD and LL wrote the manuscript. All the authors discussed the results and commented on the manuscript.

## Conflict of Interest

The authors declare that the research was conducted in the absence of any commercial or financial relationships that could be construed as a potential conflict of interest.

## Footnotes

1 https://blast.ncbi.nlm.nih.gov/Blast.cgi

2 https://www.ebi.ac.uk/Tools/msa/clustalo

3 http://unafold.rna.albany.edu/?q=mfold

4 http://weblogo.berkeley.edu/logo.cgi

5 https://www.megasoftware.net/
