# Supplementary material for: Complex RNA Secondary Structures Mediate Mutually Exclusive Splicing of Coleoptera Dscam1
Source: Front Genet. 2021 Mar 30;12:644238. doi: 10.3389/fgene.2021.644238 (PMC8042237; doi:10.3389/fgene.2021.644238)
Supplement: Supplementary file 1 [file Data_Sheet_1.pdf]

Supplementary Material

Class	Order	Family	Genus	Species	GenBank	Exon 4 copy	Exon 6 copy	Exon 9 copy
Insecta	Coleoptera	Curculionidae	*Sitophilus*	*S. oryzae* (Sor)	PPTJ02000910	10	38	36
Insecta	Coleoptera	Curculionidae	*Rhynchophorus*	*R. ferrugineus* (Rfe)	JABAOJ010000002<br>JABAOJ010000009	11	---	46
Insecta	Coleoptera	Curculionidae	*Dendroctonus*	*D. ponderosae* (Dpo)	APGL01022104<br>APGL01022105<br>APGL01022106<br>APGL01022109<br>APGL01022110<br>APGL01022112	11	26	24
Insecta	Coleoptera	Curculionidae	*Hypothenemus*	*H. hampei* (Hha)	LBGY01005917	11	21	26
Insecta	Coleoptera	Chrysomelidae	*Callosobruchus*	*C. maculatus (Cma)*	CAACVG010007189<br>CAACVG010009566	9	---	41
Insecta	Coleoptera	Cerambycidae	*Anoplophora*	*A. glabripennis* (Agl)	AQHT01042234<br>AQHT02012853<br>AQHT01017747	9	31	37
Insecta	Coleoptera	Chrysomelidae	*Leptinotarsa*	*L. decemlineata* (Lde)	AYNB02005101<br>AYNB02005102<br>AYNB02005103<br>AYNB02005104<br>AYNB02005105<br>AYNB02005107<br>AYNB02005108<br>AYNB02005109<br>AYNB02005110	8	30	28
Insecta	Coleoptera	Nitidulidae	*Aethina*	*A. tumida* (Atu)	MRBJ01000181	9	36	41
Insecta	Coleoptera	Tenebrionidae	*Tribolium*	*T. castaneum* (Tca)	AAJJ02003609	9	28	31
Insecta	Coleoptera	Silvanidae	*Oryzaephilus*	*O. surinamensis* (Osu)	SSSI01003176	9	26	28
Insecta	Coleoptera	Coccinellidae	*Coccinellini*	*C. septempunctata* (Cse)	BHEC01000016	9	24	41
Insecta	Coleoptera	Coccinellidae	*Coccinellini*	*H. axyridis* (Hax)	BHEF01029642	9	25	46
Insecta	Coleoptera	Scarabaeidae	*Onthophagus*	*O. taurus* (Ota)	HOM02010581	9	36	38
Insecta	Coleoptera	Silphidae	*Nicrophorus*	*N. vespilloides* (Nve)	LJCH01001402	9	31	53

Supplementary Table 1. Species used about the exon 4, 6, and 9 clusters of *Dscam1* in this study. All the species are listed according to class, order, family, genus, and species. The abbreviations of the species are in parentheses after the name of species. And the Genbank ID of genomic sequences are shown. The number of variable exon 4, 6, and 9 of each species is written on the right. Due to the genomic sequence break in the database, we cannot determine the number of the exon 6s of *Dscam1* in *C. maculatus* and *R. ferrugineus*.

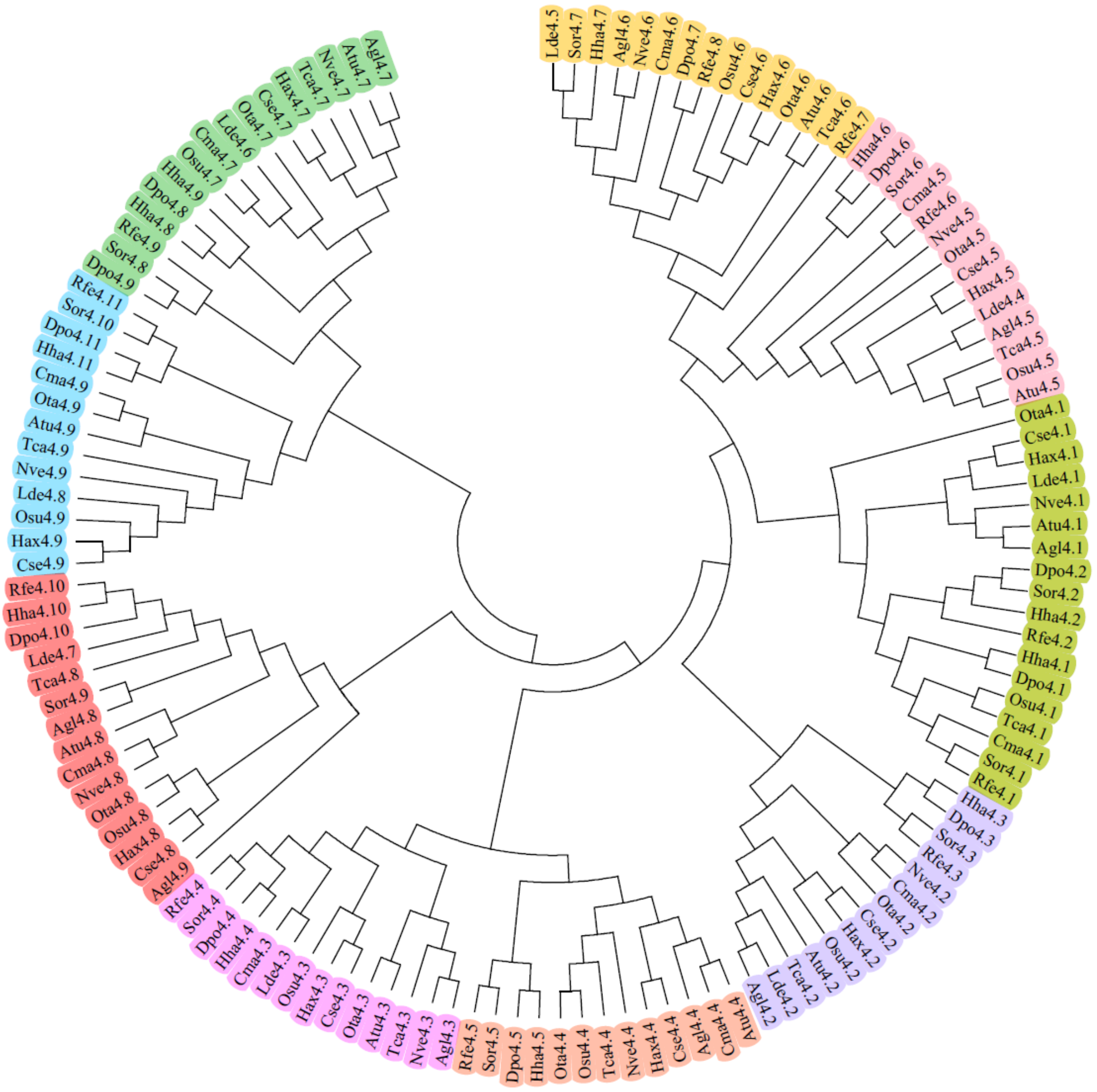

Supplementary Figure S1. The evolutionary relationship comparison of the variable exon 4s in Coleoptera species. Variable exons in the same evolutionary branch are marked with the same color. Each clade has one or two variable exons of different species which showed a one-to-one correspondence and linear order. Therefore, we hold the opinion that most exon 4s are orthologous.

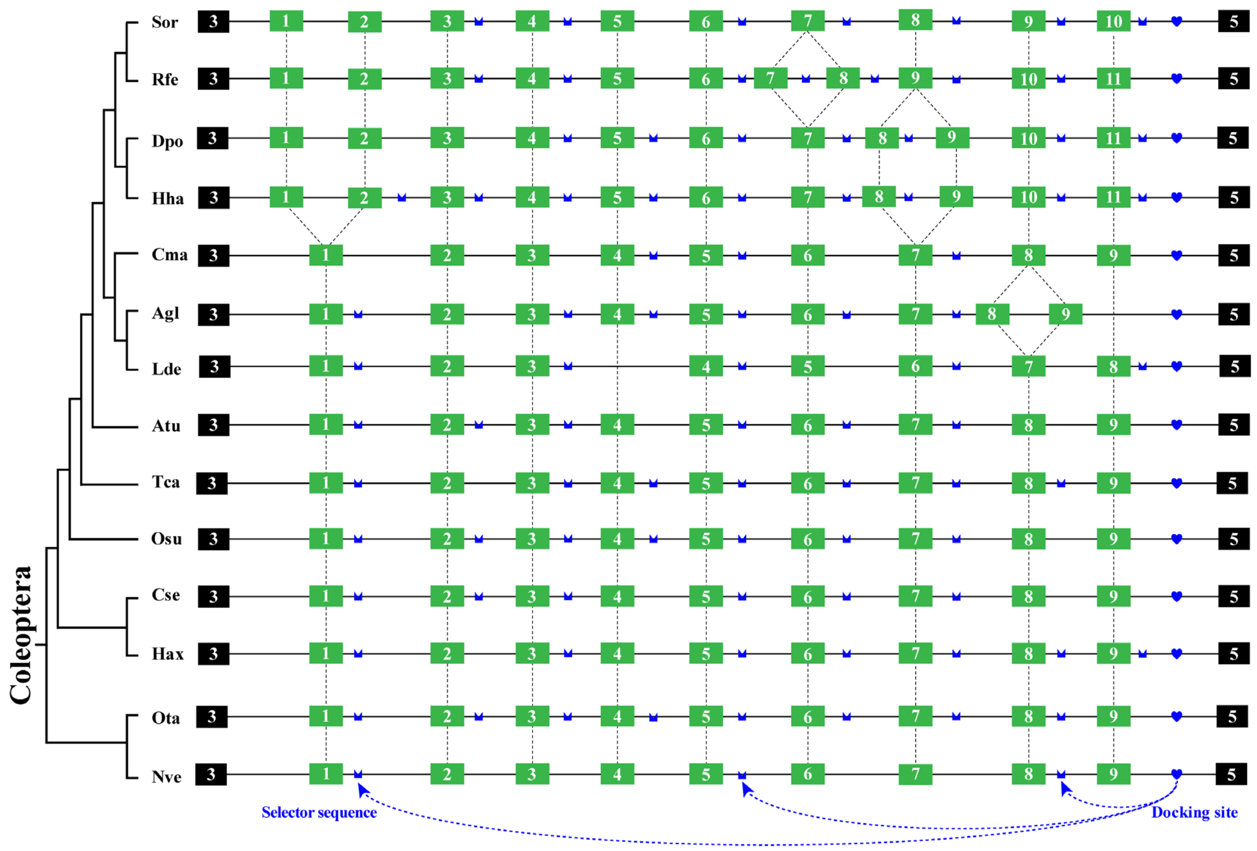

Supplementary Figure S2. RNA pairings mediate the splicing of the variable exon 4. The evolutionary relationship of variable exon 4 of *Dscam1* in Coleoptera species. Black boxes represent constitutive exons and green boxes represent variable exon 4. Exons on the same dashed line represent evolutionary relationships on the same branch. The blue heart represents the docking site of the exon 4 cluster and the blue crowns represent the selector sequences paired with the docking site.

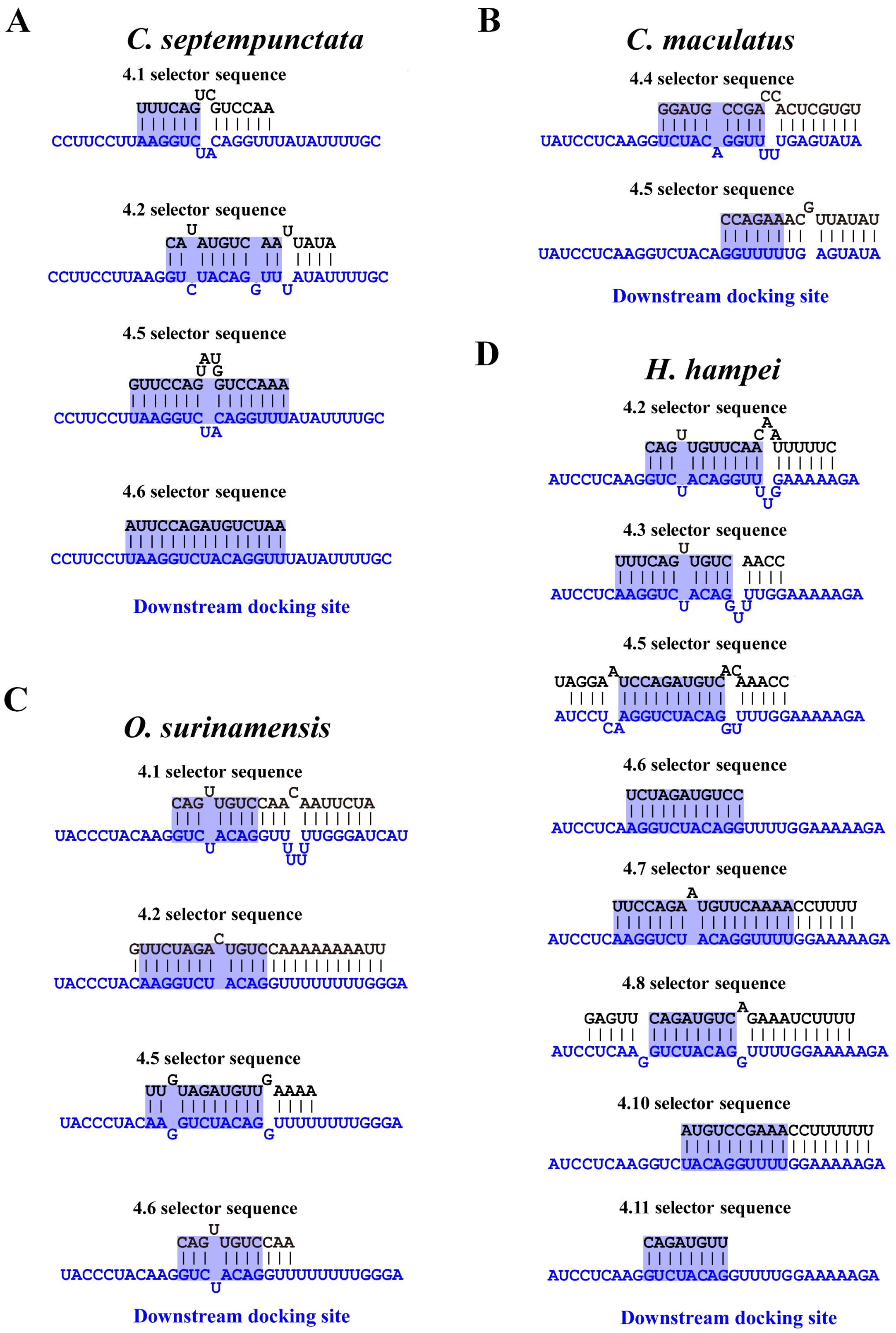

Supplementary Figure S3. Downstream base-pairings of exon 4 clusters in *C. septempunctata, C. maculatus, O. surinamensis,* and *H. hampei*. Predicted RNA-RNA interactions between docking sites and selector sequences are shown. The selector sequences are shown in black font, and the docking sites are shown in blue font. The sequences that make up the core regions of the RNA secondary structures are highlighted in blue.

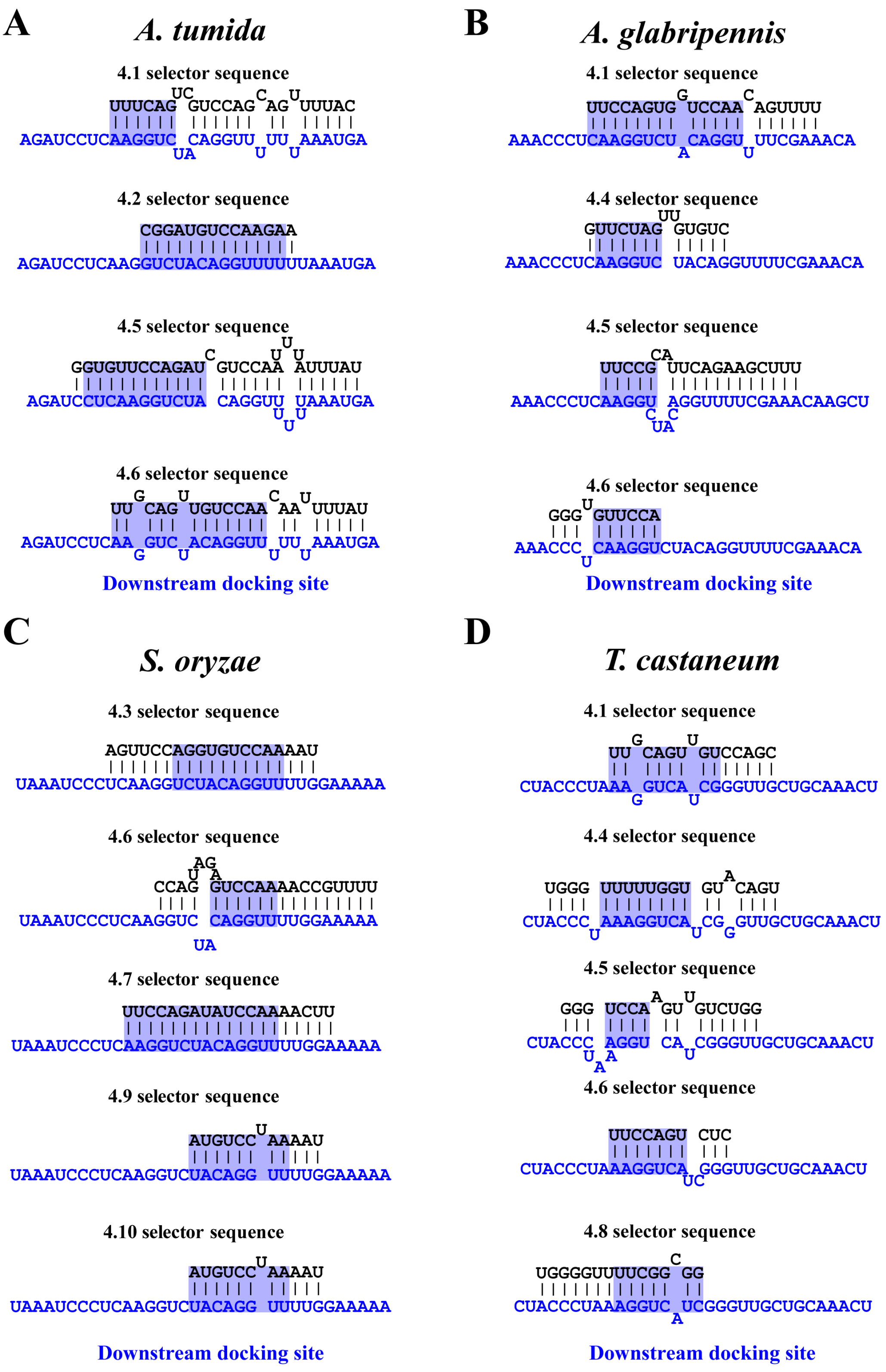

Supplementary Figure S4. RNA-RNA interactions of exon 4 cluster in *A. tumida, A. glabripennis, S. oryzae,* and *T. castaneum.* Predicted RNA secondary structure between the docking sites and selector sequences are shown. The selector sequences are shown in black font, and the docking sites are shown in blue font. The sequences that make up the core regions of the RNA secondary structures are highlighted in blue.

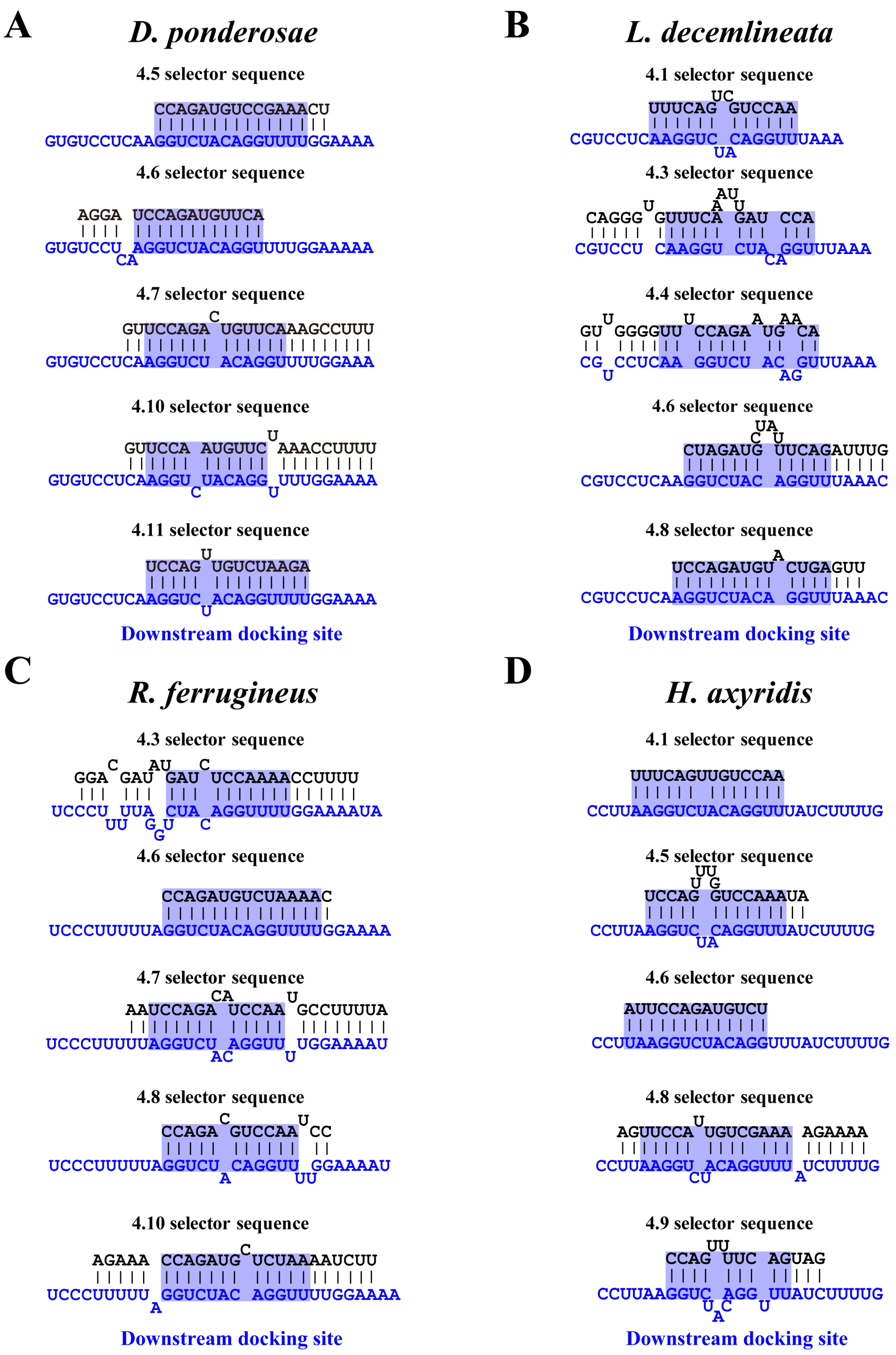

Supplementary Figure S5. Downstream base-pairing of exon 4 cluster in *D. ponderosae, L. decemlineata, R. ferrugineus,* and *H. axyridis.* Predicted RNA-RNA interactions between the docking sites and selector sequences are shown. The selector sequences are shown in black font, and the docking sites are shown in blue font. The sequences that make up the core regions of the secondary structures are highlighted in blue.

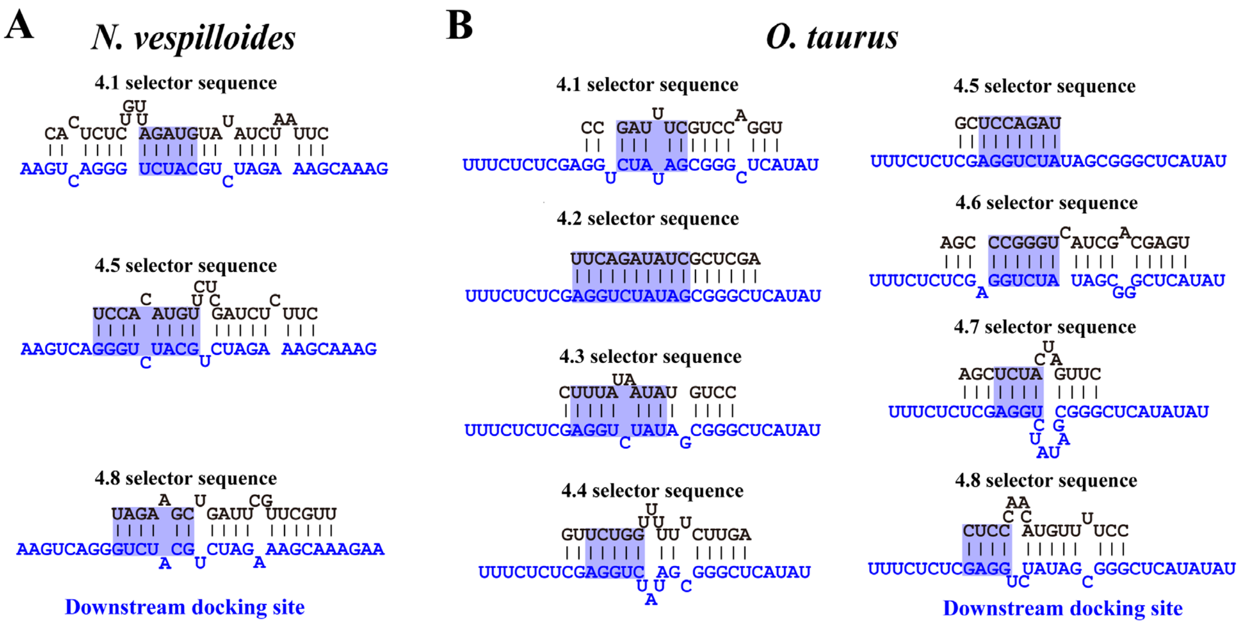

Supplementary Figure S6. RNA secondary structure of exon 4 cluster in *N. vespilloides* and *O. taurus*. Predicted RNA secondary structure interactions between docking sites and selector sequences are shown. The selector sequences are shown in black font, and the docking sites are shown in blue font. The sequences that make up the core regions of the secondary structures are highlighted in blue.

A. tumida

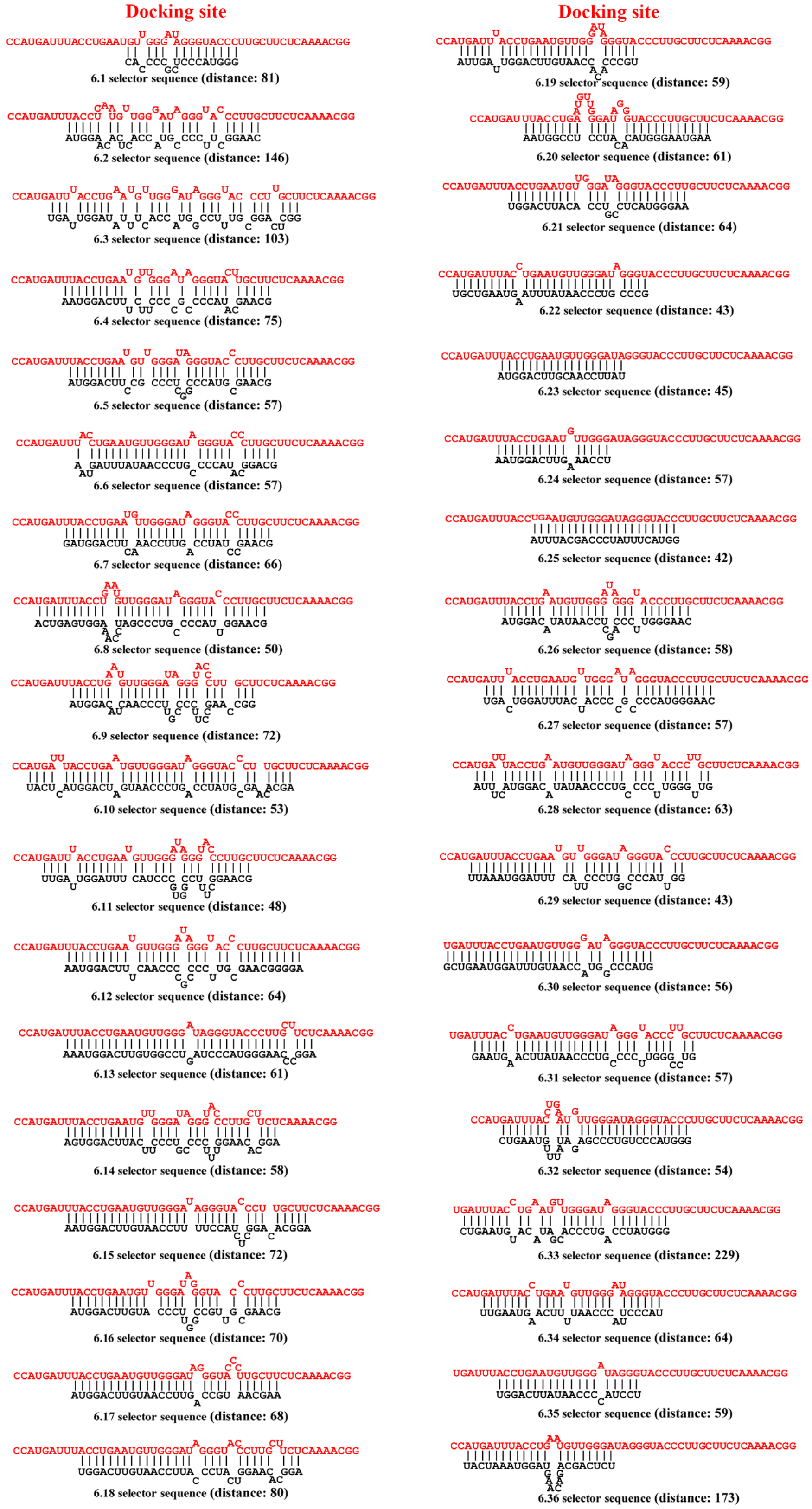

Supplementary Figure S7. Predicted RNA secondary structure of exon 6 cluster in *A. tumida*. Docking site-selector base-pairings widely exist in the exon 6 cluster. The selector sequences are shown in black font, and the docking sites are shown in red font. The distance from the selector sequence to the variable exon is shown below each RNA secondary structure.

T. castaneum

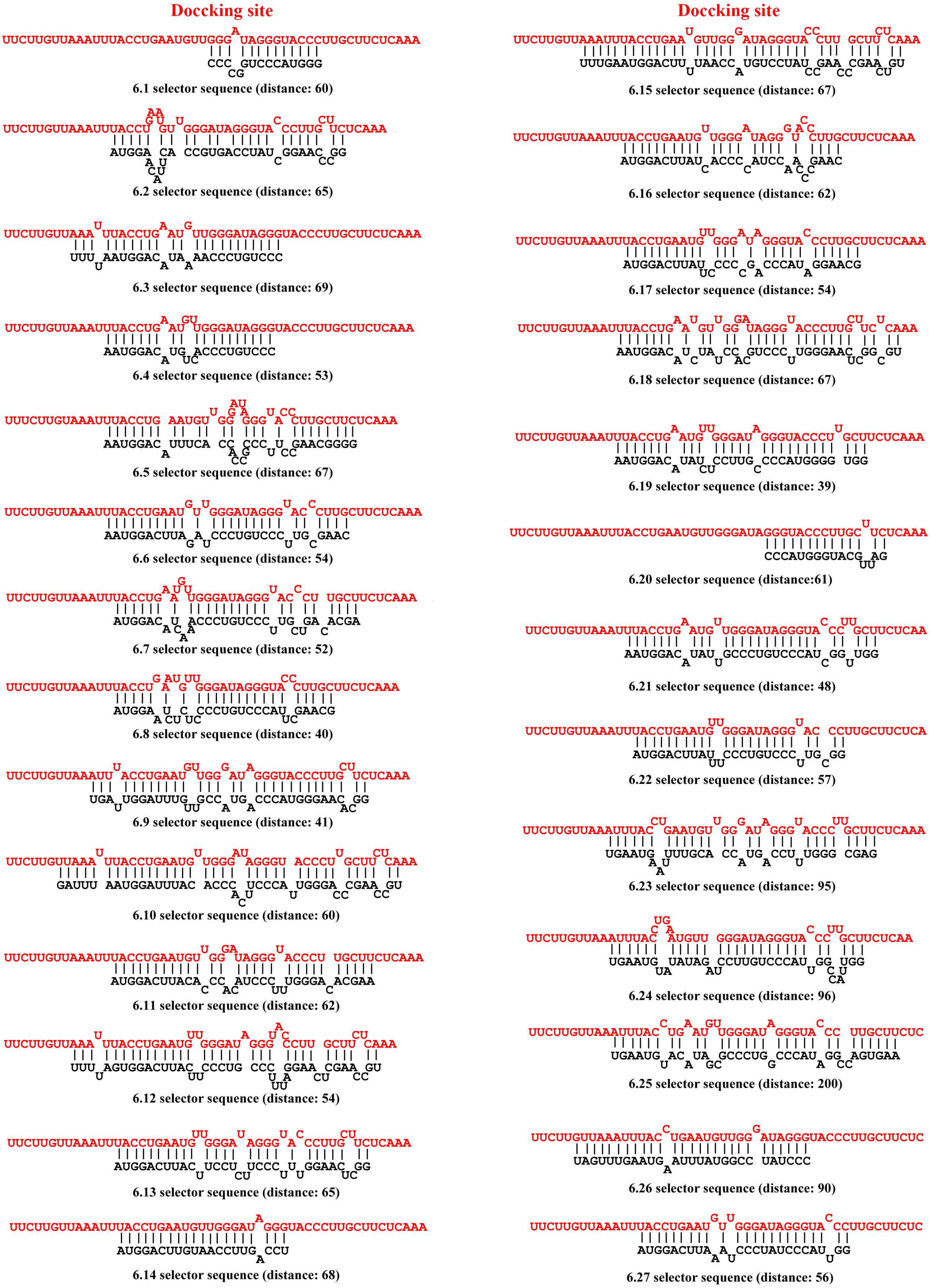

Supplementary Figure S8. Base-pairing of exon 6 cluster in *T. castaneum*. Docking site-selector base-pairings are widespread in the exon 6 cluster. The selector sequences are shown in black font, and the docking sites are shown in red font. The distance between the selector sequence and the variable exon is shown below each RNA secondary structure.

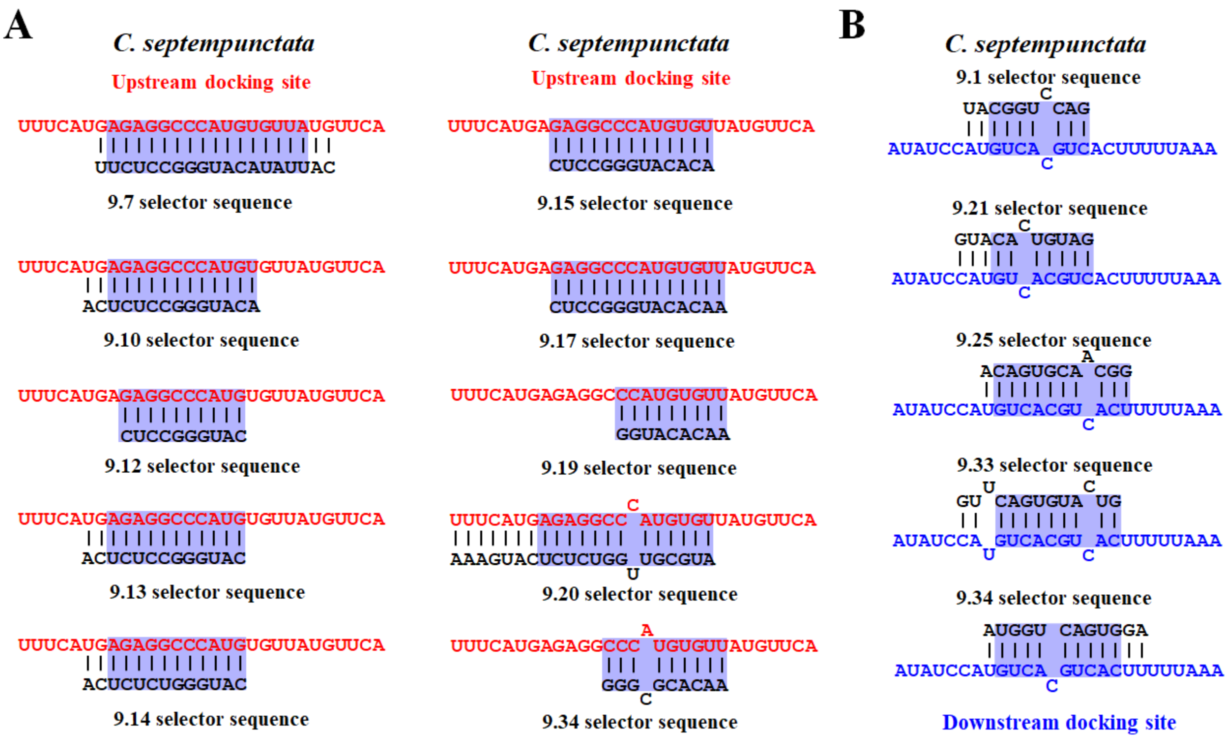

Supplementary Figure S9. Upstream and downstream base-pairings of exon 9 cluster in *C. septempunctata*. (A) The RNA secondary structures between the upstream docking site and the downstream selector sequences are shown. The core regions of RNA secondary structures are highlighted in blue. (B) The downstream base-pairings in *C. septempunctata* are shown and conserved regions of RNA secondary structures are highlighted in blue. The upstream and downstream selector sequences are shown in black font, upstream and downstream docking sites are shown in red and blue fonts, respectively.

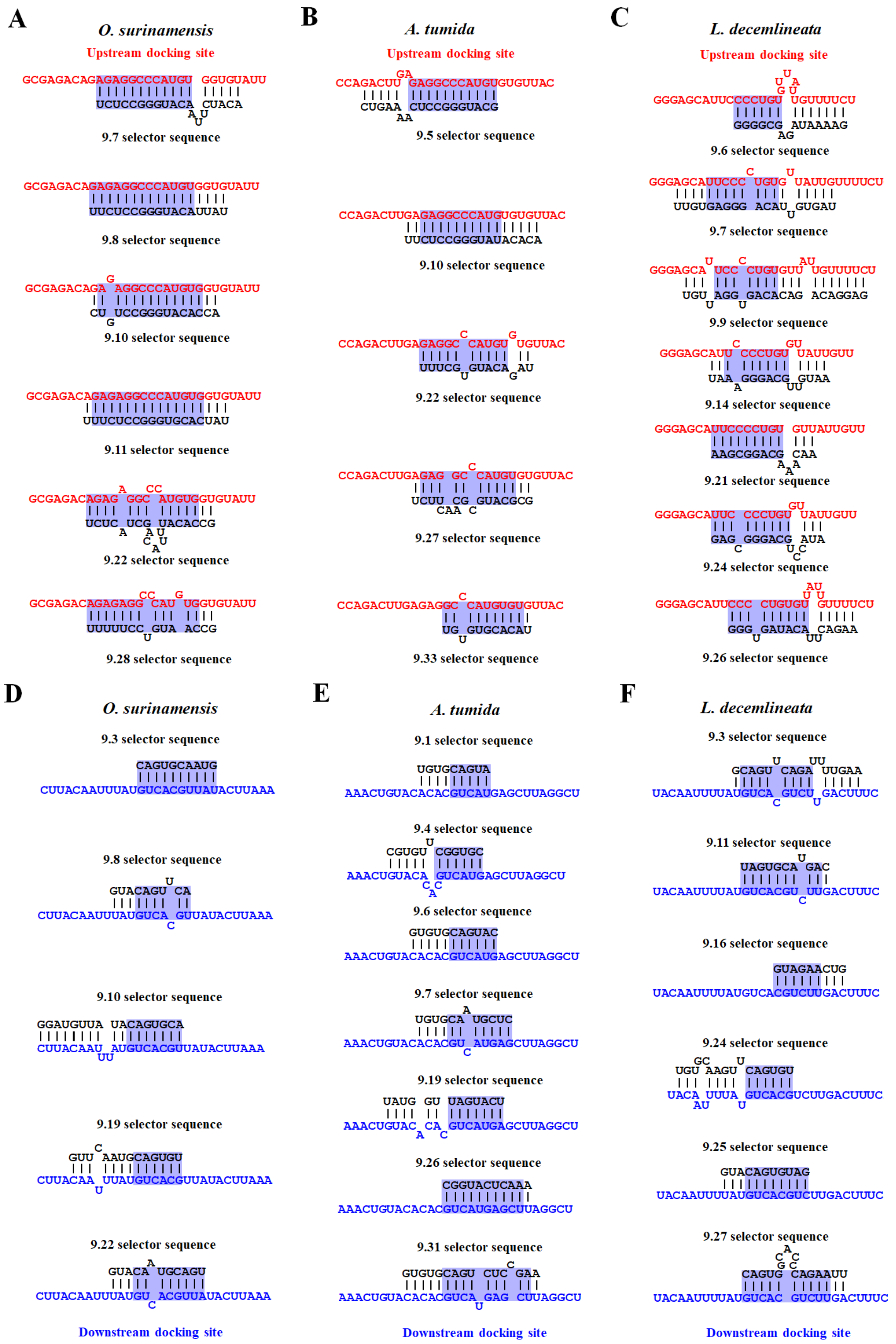

Supplementary Figure S10. Bidirectional RNA pairing of the exon 9 cluster in *O. surinamensis, A. tumida,* and *L. decemlineata*. The upstream base-pairing shows on the top and the downstream base-pairing shows on the bottom. The core regions of the secondary structures are highlighted in blue. The upstream and downstream selector sequences are shown in black font, upstream and downstream docking sites are shown in red and blue fonts, respectively.

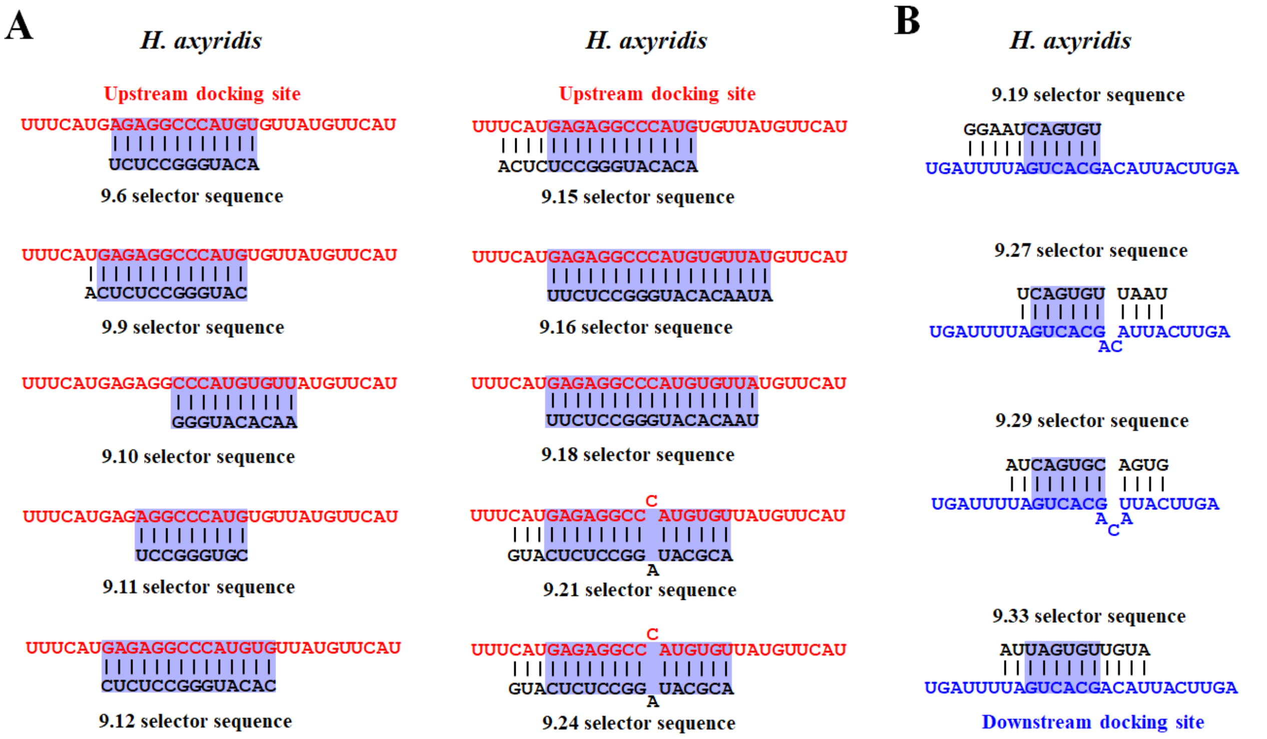

Supplementary Figure S11. Upstream and downstream base-pairings of exon 9 cluster in *H. axyridis*. (A) The base-pairings between the upstream docking site and the downstream selector sequences are shown. The core regions of RNA secondary structures are highlighted in blue. (B) The downstream base-pairings are shown in *H. axyridis* and conserved regions of secondary structures are highlighted in blue. The upstream and downstream selector sequences are shown in black font, upstream and downstream docking sites are shown in red and blue fonts, respectively.

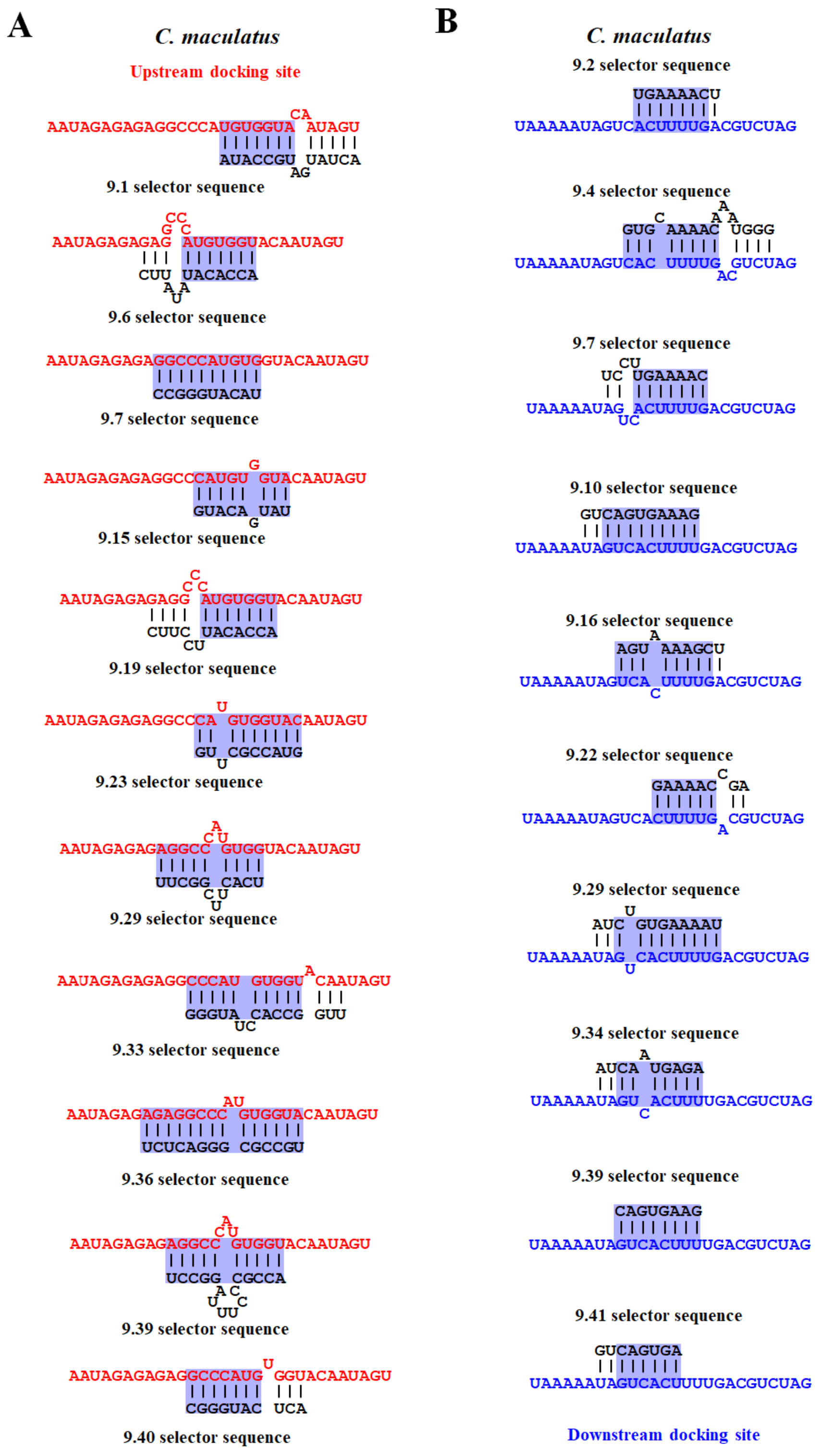

Supplementary Figure S12. Bidirectional RNA pairing of the exon 9 cluster in *C. maculatus*. (A) The secondary structures between the upstream docking site and the downstream selector sequences in *C.maculatus* are shown. The core regions of RNA secondary structures are highlighted in blue. (B)The downstream base-pairings are shown and the core regions of RNA secondary structures are highlighted in blue. The upstream and downstream selector sequences are shown in black font, upstream and downstream docking sites are shown in red and blue fonts, respectively.

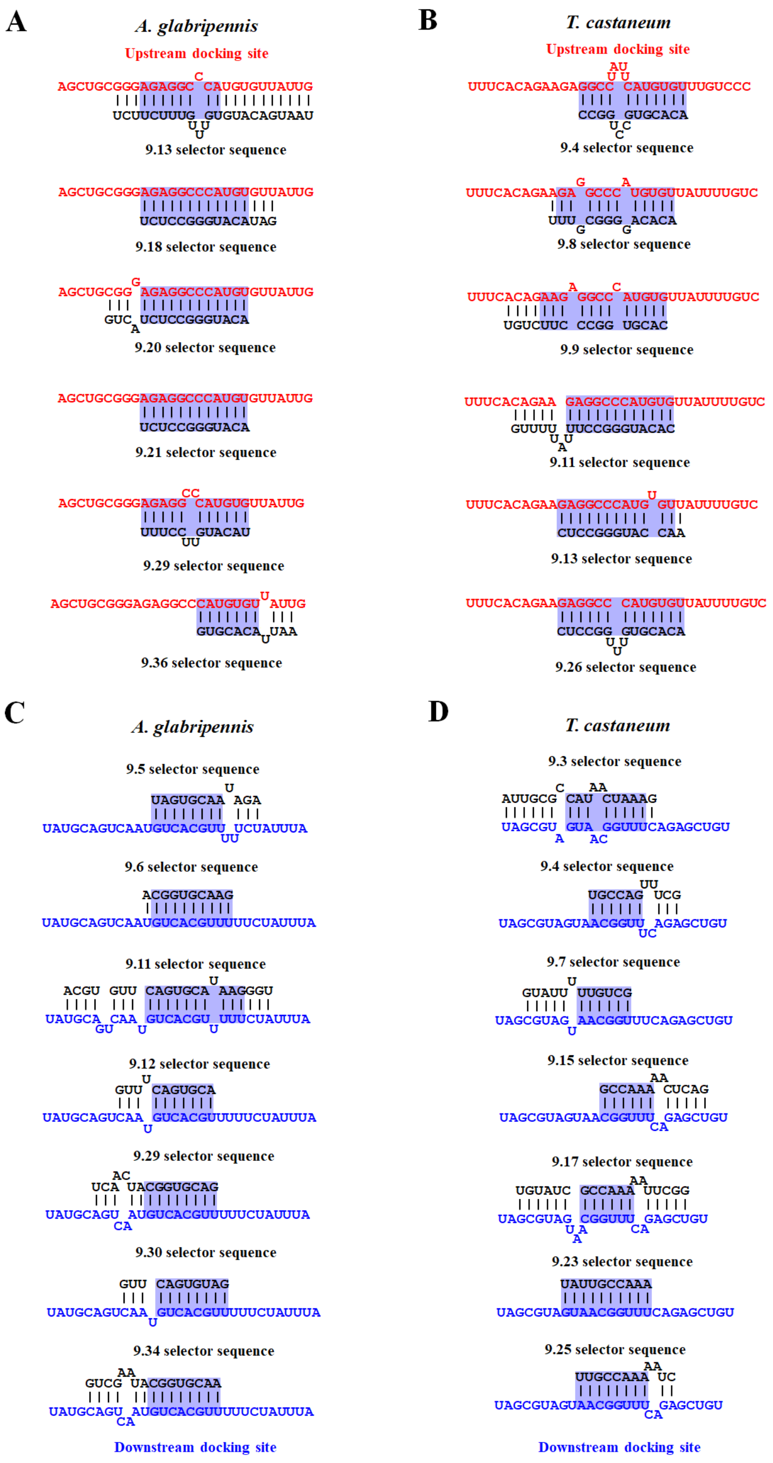

Figure S13. Upstream and downstream base-pairings of exon 9 cluster in *A. glabripennis* and *T. castaneum*. (A, B) The base-pairings between the upstream docking site and the downstream selector sequences are shown. The core regions of secondary structures are highlighted in blue. (C, D) The downstream secondary structures in *A. glabripennis and T. castaneum* are shown and conserved regions of secondary structures are highlighted in blue.

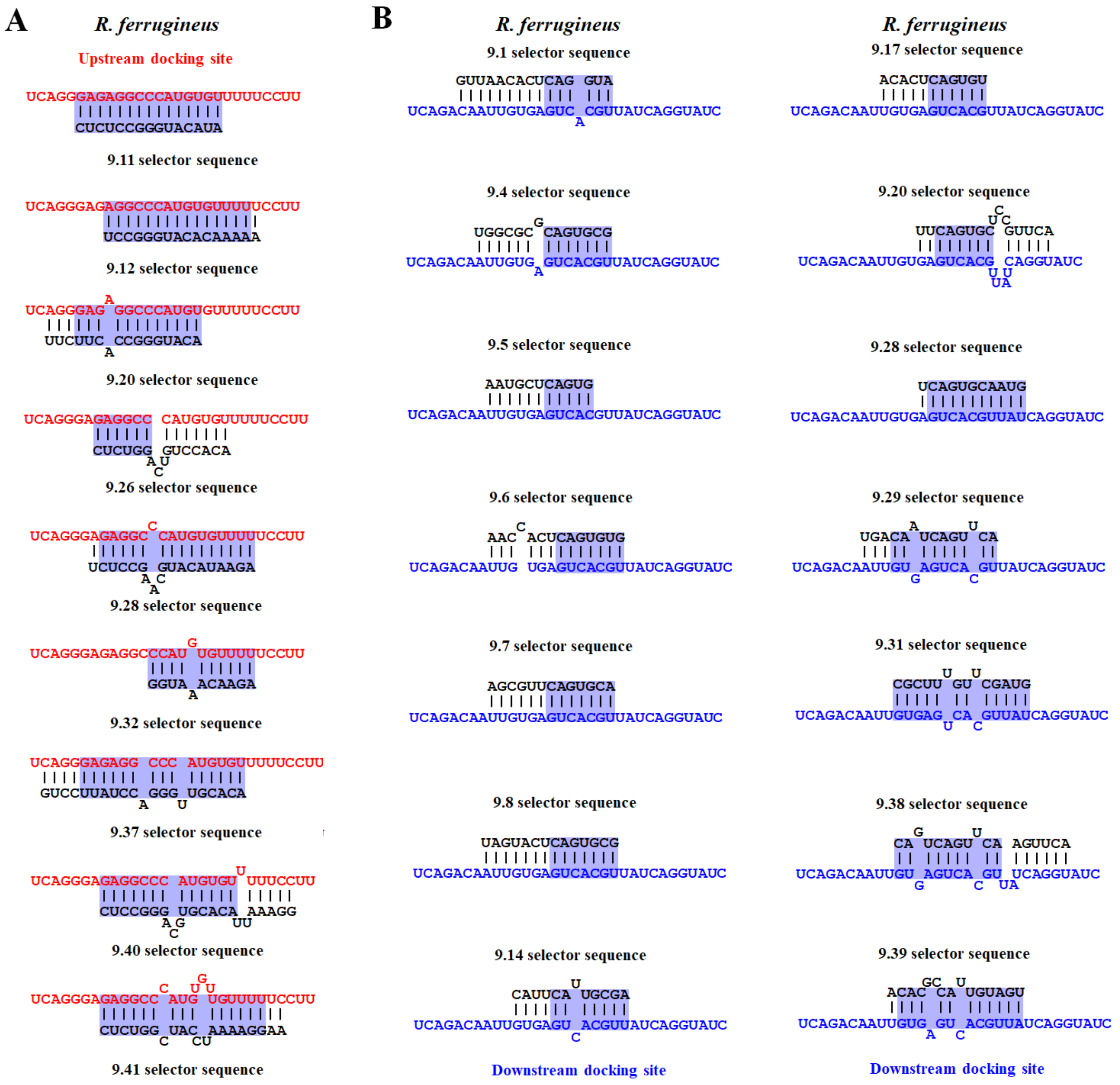

Supplementary Figure S14. Bidirectional RNA pairing of the exon 9 cluster in *R. ferrugineus*. (A) The secondary structures between the upstream docking site and the downstream selector sequences in *R. ferrugineus* are shown. The core regions of secondary structures are highlighted in blue. (B) The downstream base-pairings are shown and the core regions of secondary structures are highlighted in blue. The upstream and downstream selector sequences are shown in black font, upstream and downstream docking sites are shown in red and blue fonts, respectively.

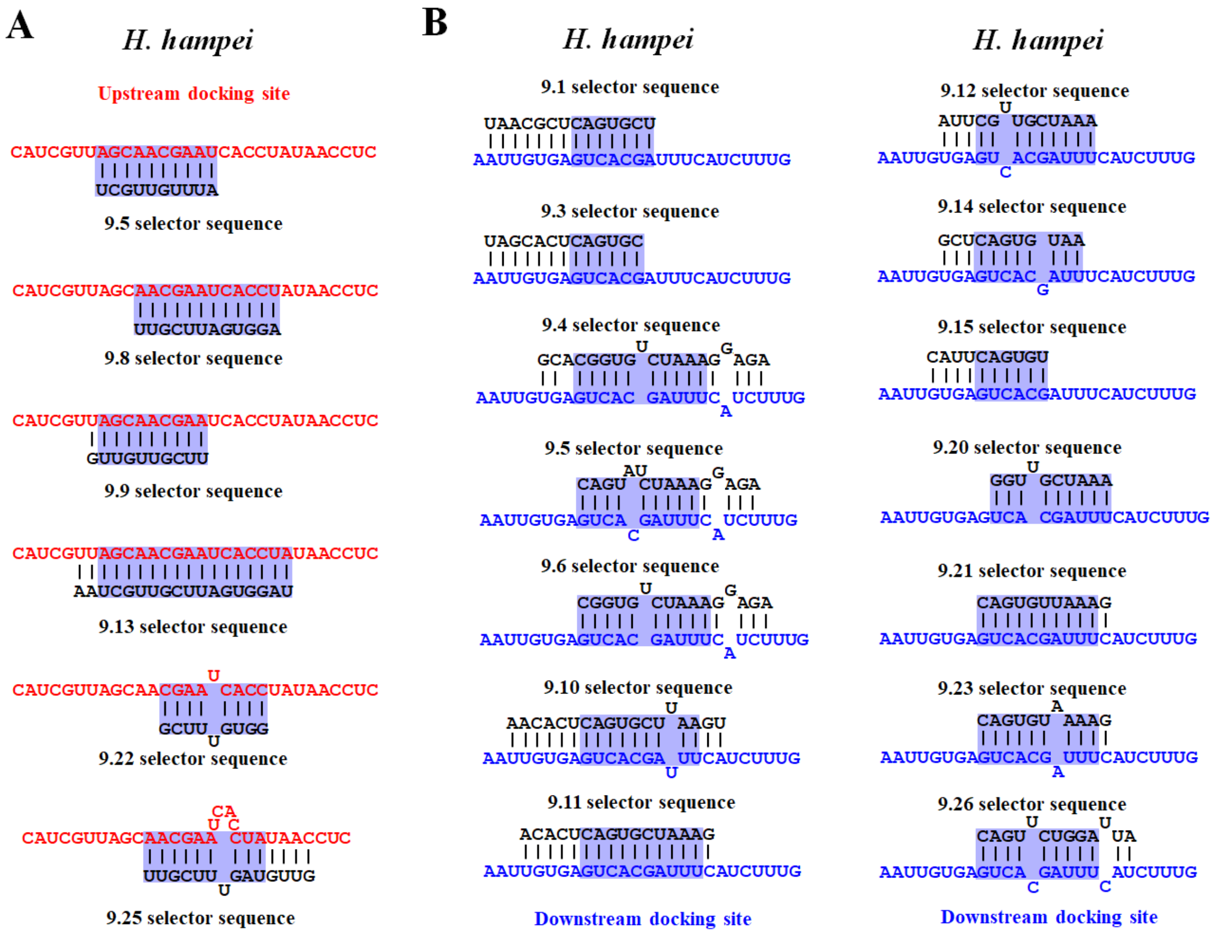

Supplementary Figure S15. Upstream and downstream base-pairings of the exon 9 cluster in *H. hampei*. (A) The base-pairings between the upstream docking site and the downstream selector sequences are shown. The core regions of secondary structures are highlighted in blue. (B) The downstream secondary structures in *H. hampei* are shown and conserved regions of secondary structures are highlighted in blue. The upstream and downstream selector sequences are shown in black font, upstream and downstream docking sites are shown in red and blue fonts, respectively.

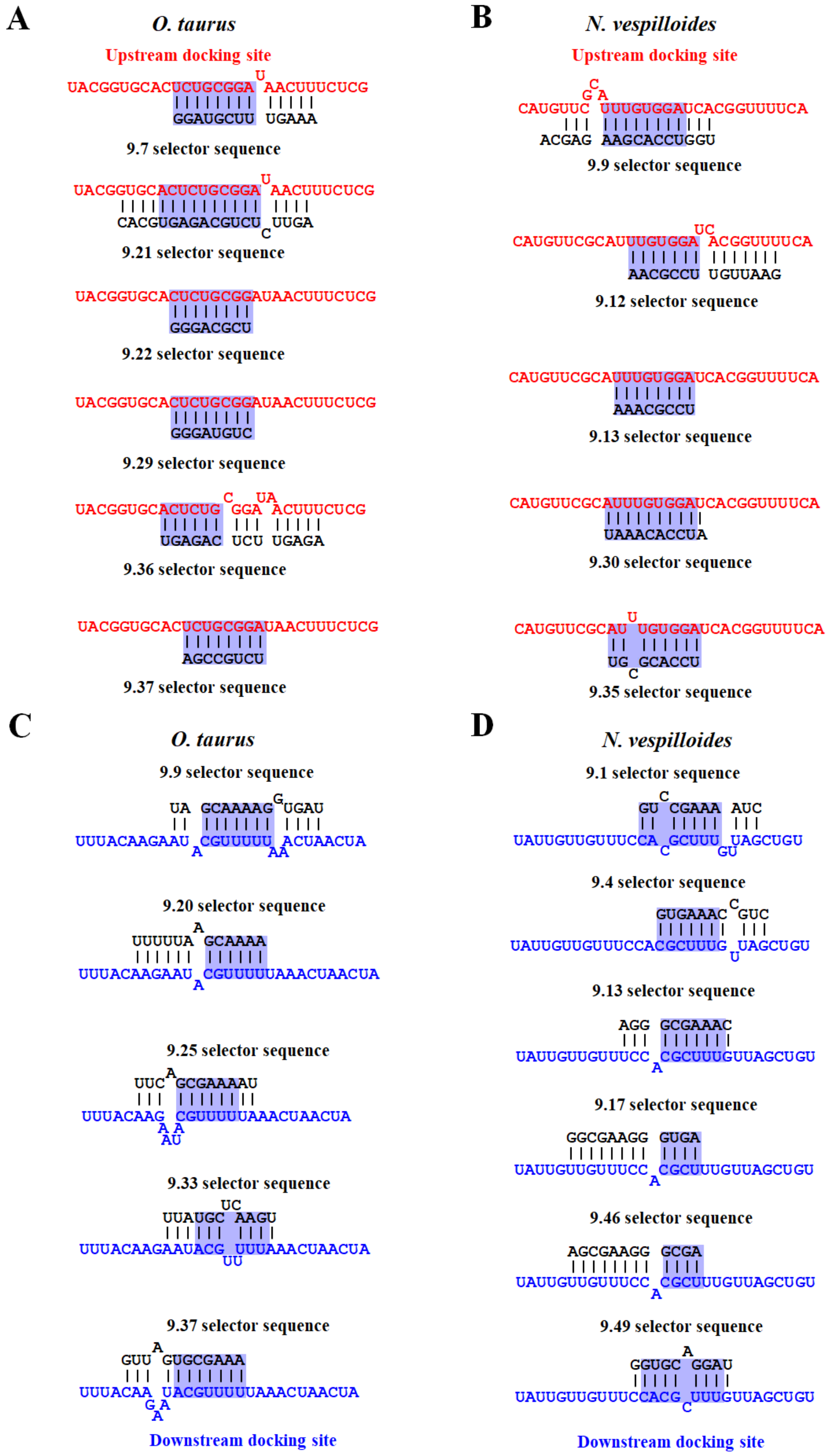

Supplementary Figure S16. Upstream and downstream base-pairings of exon 9 cluster in *O. taurus* and *N. vespilloides*. (A, B) The secondary structures between the upstream docking site and the downstream selector sequences in *O. taurus, N. vespilloides* are shown. The core regions of secondary structures are highlighted in blue. (C, D) The downstream base-pairings are shown and the core regions of secondary structures are highlighted in blue. The upstream and downstream selector sequences are shown in black font, upstream and downstream docking sites are shown in red and blue fonts, respectively.

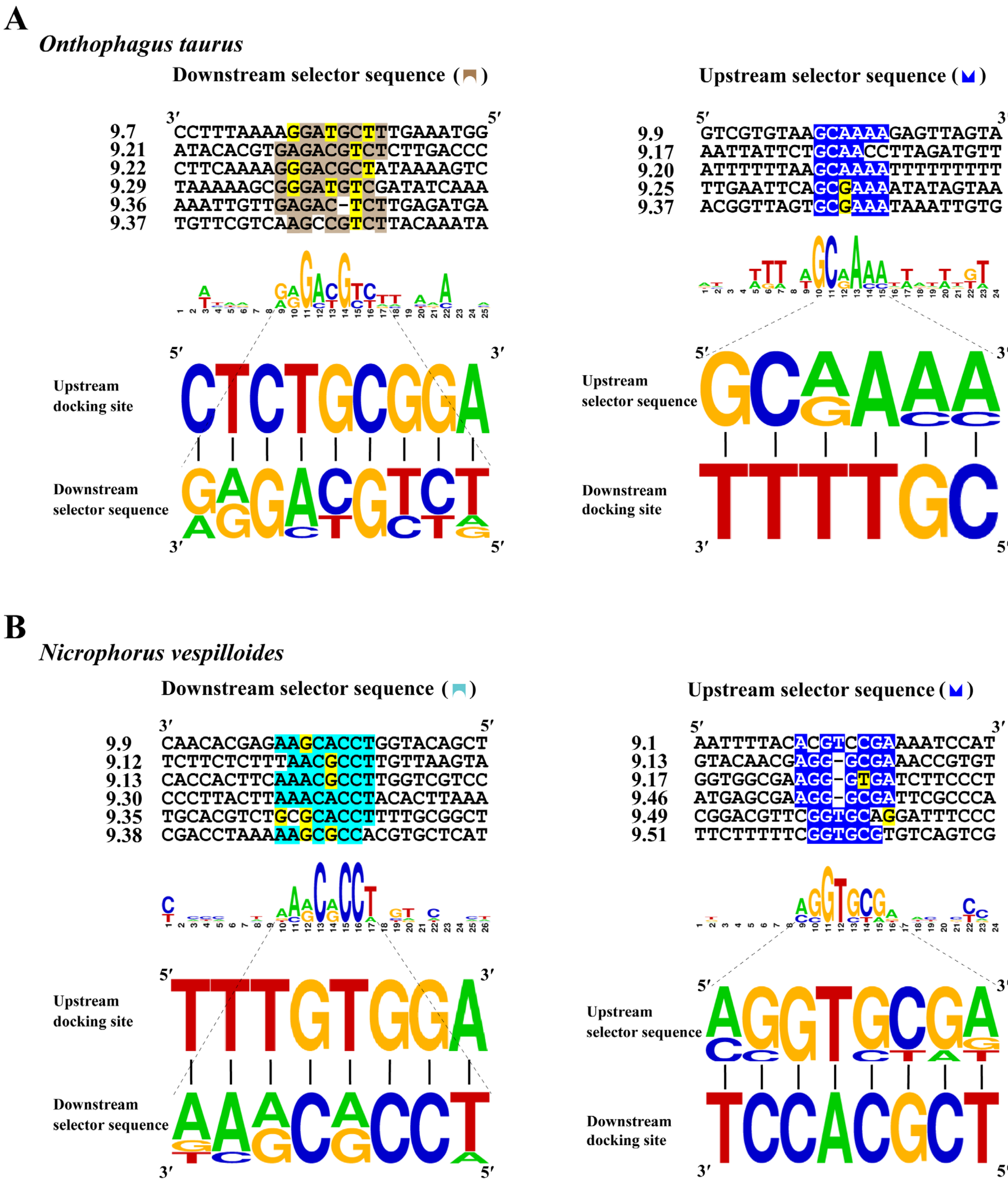

Supplementary Figure S17. Species-specific docking site of exon 9 cluster in *O. taurus* and *N. vespilloide*. (A, B) Five upstream or downstream selector sequences were chosen for sequence alignment to analyze. The most frequent nucleotides are highlighted. Nucleotide of evolutionary intermediates (U-G, G-U) are shaded in yellow. The core region of the upstream or downstream selector sequence is complementary to the downstream or upstream docking site respectively.
